# High Immunogenic Cuproptosis Evoked by In Situ Sulfidation‐Activated Pyroptosis for Tumor‐Targeted Immunotherapy of Colorectal Cancer

**DOI:** 10.1002/smsc.202300164

**Published:** 2024-01-17

**Authors:** Wentao Xiao, Kuiming Qu, Wei Zhang, Lunhui Lai, Lei He, Fang Cheng, Lianhui Wang

**Affiliations:** ^1^ State Key Laboratory for Organic Electronics and Information Displays & Jiangsu Key Laboratory for Biosensors Institute of Advanced Materials (IAM) Nanjing University of Posts and Telecommunications Nanjing 210023 P. R. China

**Keywords:** cuproptosis, immunotherapy, in situ sulfidation, MOF-199, pyroptosis

## Abstract

Despite the great potential of cuproptosis in tumor therapy, the current cuproptosis‐based therapy still suffers from compromised efficiency of immune activation. Pyroptosis, a proinflammatory cell death modality, provides a good opportunity to induce immunogenic cell death (ICD) and promote systemic immune response. However, the synergistic cuproptosis and pyroptosis therapy has not been fully explored. Herein, it is discovered that Cu(II)‐based metal–organic framework (MOF) nanoparticles (NPs) can synergistically induce cuproptosis and pyroptosis to evoke ICD for high‐efficiency tumor‐targeted immunotherapy. Although MOF‐199 has been widely used in tumor therapy, the immunogenicity is still unclear. Pluronic F127‐modified MOF‐199 NPs (^F127^MOF‐199 NPs) show dual‐responsiveness to glutathione (GSH) and hydrogen sulfide (H_2_S). Once entering cancer cells, ^F127^MOF‐199 NPs dissociate in GSH‐enriched tumor microenvironment (TME) to release copper ion and induce copper‐overload‐mediated cuproptosis. Meanwhile, ^F127^MOF‐199 NPs transform to Cu_2−*x*_S NPs by in situ sulfidation under H_2_S‐enriched colorectal cancer (CRC) TME. Under photothermal and chemodynamic therapy (PTT/CDT) of Cu_2−*x*_S NPs, caspase‐3 is activated and gasdermin E (GSDME)‐related pyroptosis is triggered. The synergistic cuproptosis and pyroptosis have proved the superior antitumor immunity effect in both in vitro and in vivo experiments. This work provides a new strategy to achieve tumor‐targeted immunotherapy with high efficiency by simple ^F127^MOF‐199 NPs.

## Graphical abstract

MOF‐199 nanoparticles (NPs) with dual‐responsiveness to the tumor microenvironment (TME) are developed as nanoinducers to evoke high immunogenic cuproptosis through in situ sulfidation‐activated pyroptosis. The induced immunogenic cell death (ICD) exhibits a superior antitumor immunity effect for tumor‐targeted therapy of colorectal cancer (CRC).

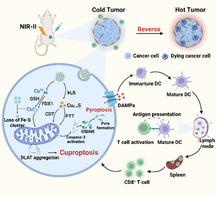

## 1 Introduction

Colorectal cancer (CRC), the second lethality malignant tumor, poses a serious threat to human health.^[^ ^1^ ^]^ Despite of the rapid development in diagnosis and treatment, metastases still occur in approximately 50% of patients.^[^ ^2^ ^]^ Immunotherapy has emerged as a promising approach against metastases by using patients’ immunity system to combat with tumor.^[^ ^3^ ^]^ Unfortunately, the majority of CRC patients show poor response to immunotherapy since CRC is a typical “cold tumor” with low immunogenetic.^[^ ^4^ ^]^ Inducing immunogenic cell death (ICD) is an effective way to improve the sensitivity to immunotherapy.^[^ ^5^ ^]^ ICD elicits the release of antigens and danger associated molecular patterns (DAMPs), such as calreticulin (CRT), high mobility group box 1 (HMGB1) and adenosine triphosphate (ATP), to activate the adaptive immune system and reverse “cold tumor” to “hot tumor”.^[^ ^6^ ^]^ However, the efficient ICD is mostly induced through apoptosis, and the therapeutic effect is still far from satisfied due to the intrinsic tumor resistance to apoptosis.^[^ ^7^ ^]^ Therefore, developing novel cancer cell death modalities with high immunogenicity is urgently required.

Cuproptosis is a new form of programmed cell death (PCD), characterized by copper overload induced aggregation of mitochondrial lipoylated proteins and destabilization of Fe–S cluster proteins.^[^ ^8^ ^]^ As the cofactor of various enzymes, copper plays an important role in various biochemical processes, including antioxidant defense, mitochondrial respiration, and biosynthesis.^[^ ^9^ ^]^ The intracellular copper level is maintained by a conserved homeostasis mechanism, while the overload of copper can lead to cuproptosis.^[^ ^10^ ^]^ Due to the completely different cellular mechanism, cuproptosis can efficiently circumvent apoptosis resistance. Recently, cuproptosis pathway has attracted extensive attention in cancer therapy.^[^ ^11^ ^]^ Due to the high Fenton catalytic efficiency, copper ion could produce reactive oxygen species (ROS)‐dependent endoplasmic reticulum (ER) stress to induce ICD.^[^ ^12^ ^]^ However, the ROS‐induced ICD is usually limited due to the high level of reducing substance in tumor microenvironment (TME).^[^ ^13^ ^]^

Pyroptosis, another form of PCD, is featured by gasdermin (GSDM)‐mediated membrane perforation and cell swelling, leading to the fast release of DAMPs.^[^ ^14^ ^]^ As an inflammatory pathway, pyroptosis can induce ICD and convert “cold tumor” to “hot tumor”.^[^ ^15^ ^]^ Particularly, gasdermin E (GSDME), one of the widely studied GSDM proteins, can switch apoptosis to pyroptosis and activate immune response in cell lines with high GSDME expression.^[^ ^16^ ^]^ Considering the high immunity activation efficiency of pyroptosis, developing advanced nanomaterials to synergistically induce pyroptosis and cuproptosis could be an ideal way to strengthen the immunogenicity of cuproptosis‐based cancer therapy.

A common problem of cuproptosis and pyroptosis‐based therapy is the potential damage to normal cells without targeting. Stimuli‐responsive intelligent nanomaterials, which actively target TME, can specifically kill tumor cells while sparling normal cells.^[^ ^17^ ^]^ The high level of glutathione (GSH) is a typical hallmark of TME.^[^ ^18^ ^]^ As a copper chelator, GSH can mitigate copper overload and inhibit cuproptosis.^[^ ^19^ ^]^ Moreover, GSH can reduce the immunogenicity of ROS‐induced ICD.^[^ ^20^ ^]^ Therefore, design GSH‐responsive nanomaterials to deplete intracellular GSH can not only endow tumor targeting, but also enhance cuproptosis‐medicated immunogenicity. Hydrogen sulfide (H_2_S) is usually overexpressed in CRC.^[^ ^21^ ^]^ High level of H_2_S can enhance cuproptosis by inhibiting copper exporter ATP7A with the function of pumping out excessive intracellular copper, thus H_2_S‐induced cuproptosis can be used for targeted therapy of CRC.^[^ ^22^ ^]^ More importantly, the affinity between H_2_S and copper ion can generate copper sulfide in situ for CRC‐targeted photothermal and chemodynamic therapy (PTT/CDT).^[^ ^23^ ^]^ Therefore, design H_2_S‐responsive nanomaterials is an ideal way to enhance CRC‐specific cuproptosis. The high level of GSDME in CRC (especially in CT26 cell lines) provides a great opportunity to trigger GSDME‐mediated pyroptosis.^[^ ^24^ ^]^ Taking together, it is highly desirable to develop GSH and H_2_S dual‐responsive nanomaterials to synergistically induce CRC‐specific cuproptosis and pyroptosis for immunotherapy.

**Scheme 1:**
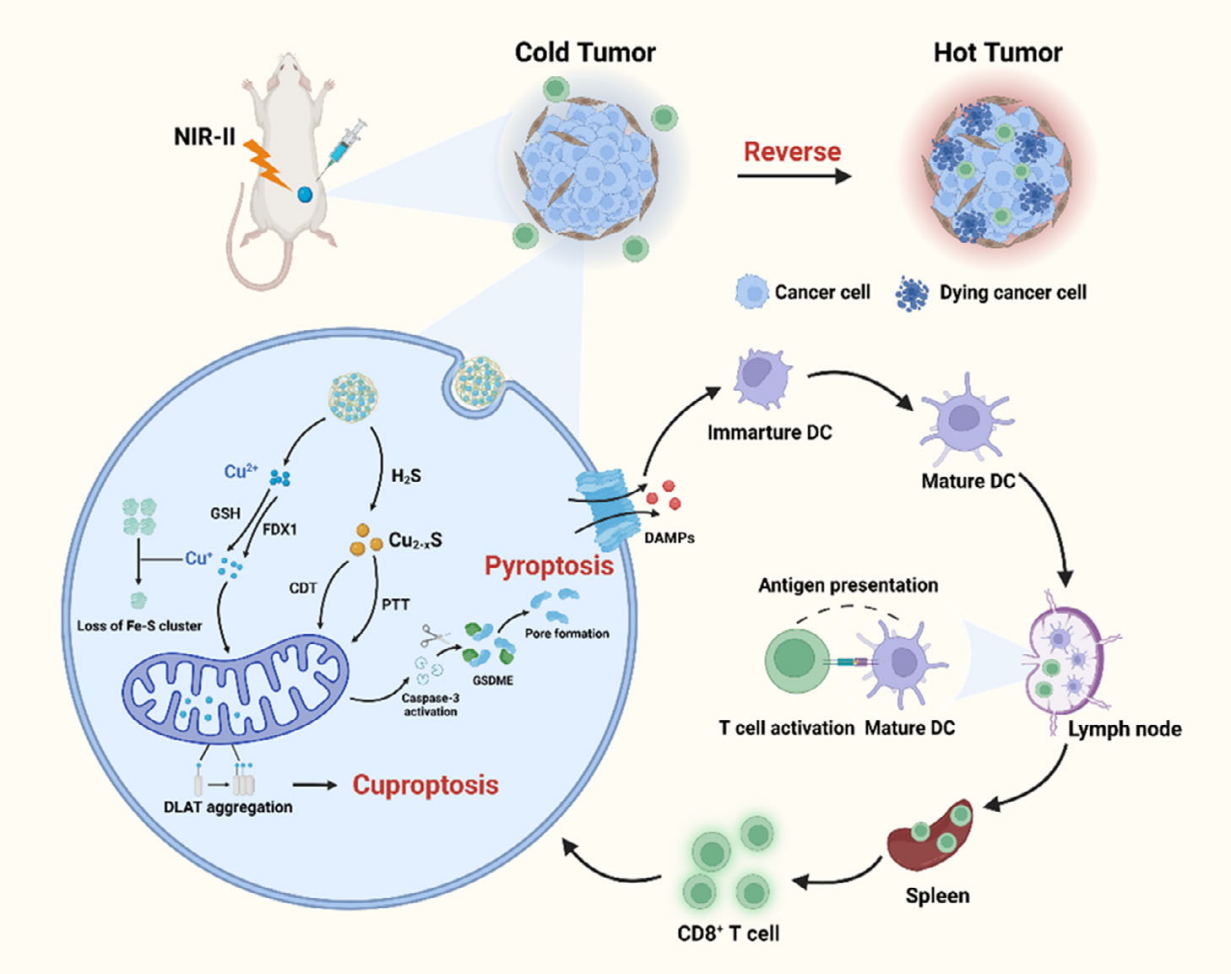
Schematic illustration of ^F127^MOF‐199 NPs served as GSH and H_2_S dual‐responsive nanomaterials for CRC‐targeted therapy based on cuproptosis and pyroptosis.

Herein, we discover that MOF‐199, a well‐studied Cu(II)‐based metal–organic framework, can synergistically induce cuproptosis and pyroptosis to efficiently evoke ICD for CRC‐specific immunotherapy. Although MOF‐199 has been used in a variety of tumor therapy,^[^ ^25^ ^]^ the immunogenicity of MOF‐199 nanoparticles (NPs) has not been fully explored. In this work, we discovered that the Pluronic F127 modified MOF‐199 NPs (^F127^MOF‐199 NPs) can response to high level of GSH and H_2_S in CRC, and further induced copper‐overload‐mediated cuproptosis and GSDME‐related pyroptosis. Once endocytosed, ^F127^MOF‐199 NPs dissociated in GSH‐rich TME to release Cu^2+^, which could further convert to more toxic Cu^+^ with GSH or FDX1. The liberated Cu^+^ can bind to lipoylated mitochondrial enzymes to induce the aggregation of lipoylated DLAT and eventually lead to cuproptosis. Meanwhile, ^F127^MOF‐199 NPs sulfated in situ to generate Cu_2*−x*_S NPs for NIR‐II PTT and CDT. The pyroptosis was triggered by synergistic PTT and CDT in GSDME high‐expressed CRC with the fast release of DAMPs, which further induce ICD for activation of immune system with high efficiency. Eventually, the synergistic cuproptosis and pyroptosis significantly enhance the immunogenic of cuproptosis‐based tumor therapy, and effectively reverse “cold tumor” to “hot tumor” (**Scheme** **1**). This study points to the great potential of MOF‐199 NPs in tumor‐targeted immunotherapy based on cuproptosis and pyroptosis.

## 2 Results and Discussions

### 2.1 Preparation and Characterization of ^F127^MOF‐199 NPs

**Figure 1:**
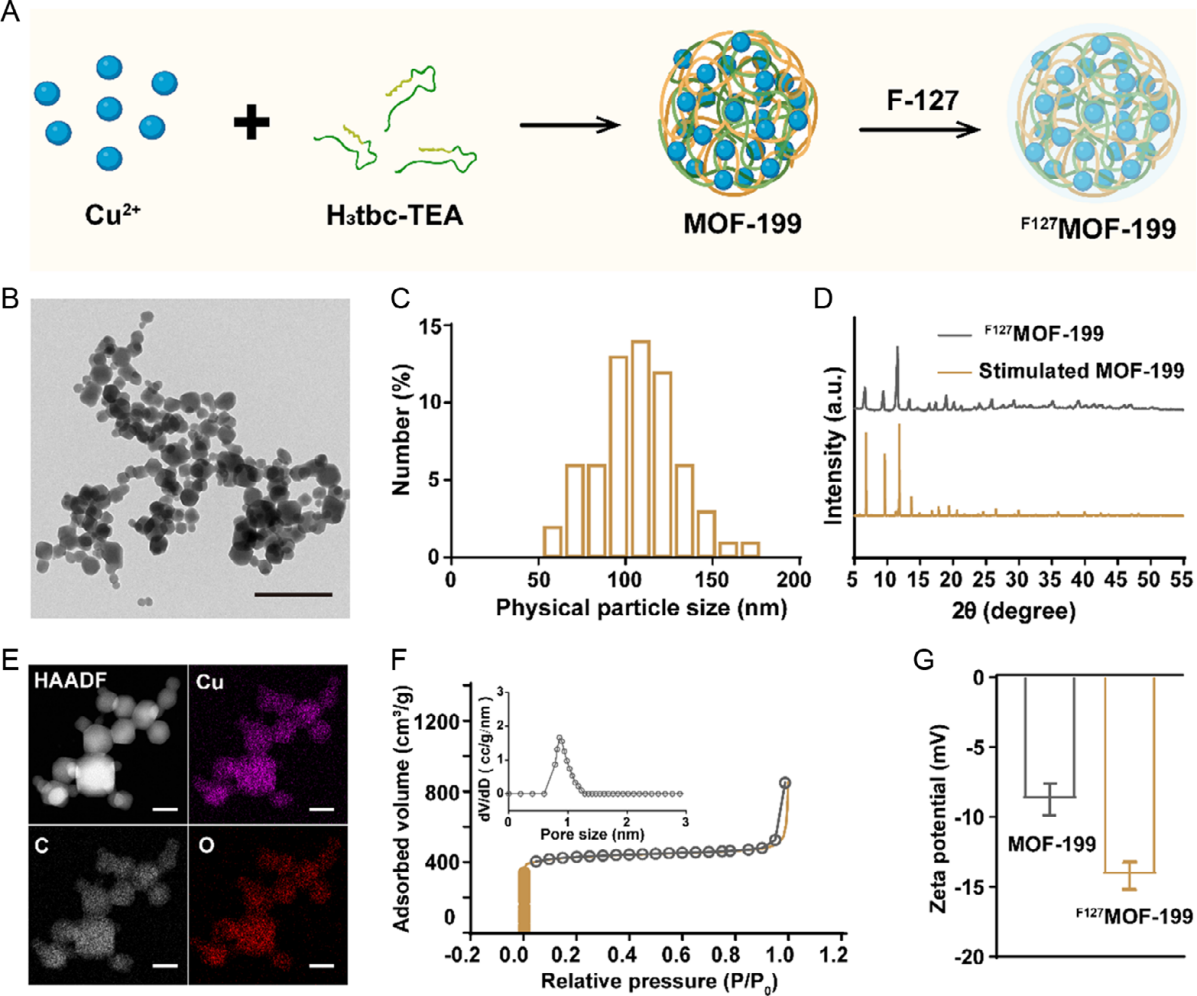
Material synthesis and characterization. A) Schematic illustration of the synthesis of ^F127^MOF‐199 NPs. B) TEM image (scale bar = 500 nm), C) size distribution, D) XRD, E) HAADF‐STEM image, EDS elemental mappings (scale bar = 50 nm), and F) N_2_ adsorption and desorption isotherms of ^F127^MOF‐199 NPs. G) Zeta potentials of MOF‐199 NPs and ^F127^MOF‐199 NPs.

Firstly, MOF‐199 NPs were synthesized by the reaction between benzene‐1,3,5‐tricarboxylate triethylammonium salt and Cu(NO_3_)_2_ in the mixture of ethanol and deionized water.^[^ ^26^ ^]^ Then, Pluronic F127 was used to encapsulate MOF‐199 NPs to improve the in vivo stability and biocompatibility (**Figure** **1**A). The transmission electron microscopy (TEM) image revealed the well‐dispersed ^F127^MOF‐199 NPs with the average size of ≈107.8 nm (Figure 1B,C). The X‐Ray diffraction (XRD) patterns of ^F127^MOF‐199 NPs displayed a good agreement with the simulated MOF‐199, indicating the high crystallinity was not affected by the surface modification (Figure 1D). The high‐angle annular dark‐field scanning TEM (HADDF‐STEM) images and the corresponding energy‐dispersive X‐ray spectroscopy (EDS) elemental mappings showed the homogenous distribution of Cu, C, and O over the whole NPs (Figure 1E). The X‐ray photoelectron spectroscopy (XPS) was conducted to reveal the chemical state of Cu in the NPs. The survey scan given in Figure S1A (Supporting Information) confirmed the presence of Cu, N, O, and C elements in the NPs. The corresponding high‐resolution XPS spectra of Cu 2p revealed the two characteristic peaks at 932.6 and 952.5 eV (Figure S1B, Supporting Information), which was in agreement with the binding energies of Cu(II)2p_3/2_ and Cu(II)2p_1/2_.^[^ ^27^ ^]^ Moreover, the satellite peaks appearing between the binding energies of 2p_3/2_ and 2p_1/2_ further confirmed the presence of Cu^2+^ in the synthesized NPs.^[^ ^28^ ^]^ The high specific surface area (1688.8 m^2^ g^−1^) and well‐defined pore size (0.8−1.2 nm) proved the porous structure of ^F127^MOF‐199 NPs (Figure 1F). The successful encapsulation of negative charged F‐127 on MOF‐199 was demonstrated by the apparently decreased zeta potential (Figure 1G). No obvious change in UV–Vis–NIR spectra after encapsulation demonstrated the property of MOF‐199 NPs was not affected by the encapsulation of F‐127 (Figure S1C, Supporting Information). The almost unchanged hydrodynamic diameter in different biological media suggested the good colloidal stability of the synthesized ^F127^MOF‐199 NPs (Figure S2, Supporting Information). The red blood cell hemolysis analysis showed negligible hemolytic activity even with high concentration of ^F127^MOF‐199 NPs (200 μg mL^−1^), indicating the excellent biocompatibility of ^F127^MOF‐199 NPs (Figure S3, Supporting Information). All these results demonstrated the successful preparation of ^F127^MOF‐199 NPs with good biocompatibility.

### 2.2 The Dual‐Responsiveness of ^F127^MOF‐199 NPs

**Figure 2:**
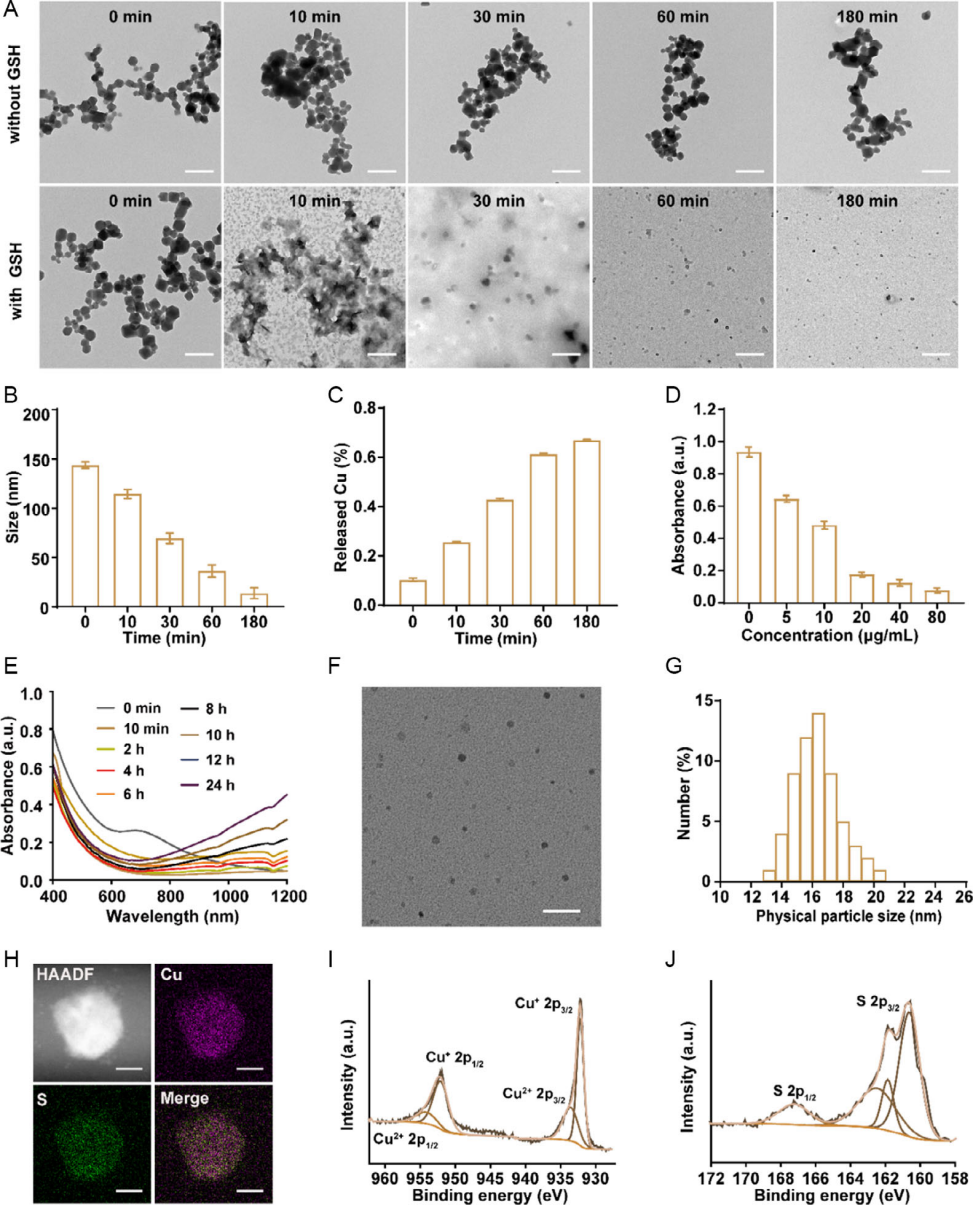
Dual‐responsiveness of ^F127^MOF‐199 NPs. A) TEM images of ^F127^MOF‐199 NPs (200 μg mL^−1^) treated with or without GSH (10 mM) for 0−180 min (scale bar = 500 nm). B) Hydrodynamic diameters and C) released Cu^2+^ for ^F127^MOF‐199 NPs treated with GSH for different time. D) GSH consumption of ^F127^MOF‐199 NPs using DTNB as the probe. E) UV–Vis–NIR absorption spectra of ^F127^MOF‐199 NPs (200 μg mL^−1^) treated with NaHS (3.0 mM) for different time. F) TEM image, G) size distribution, H) HAADF‐STEM image, EDS element mappings, and I,J) XPS spectra of ^F127^MOF‐199 NPs after treated with NaHS (scale bar in (F) = 50 nm; scale bar in (H) = 10 nm).

The GSH‐responsiveness of nanomaterials is not only critical for achieving tumor targeting, but also beneficial for sensitizing cuproptosis. Therefore, we evaluated the GSH‐induced degeneration and ion release of ^F127^MOF‐199 NPs. The TEM images displayed negligible morphology change of ^F127^MOF‐199 NPs after dispersed in PBS (pH = 7.4) for 3 h, indicating the high stability under physiological conditions. In contrast, ^F127^MOF‐199 NPs started to degenerate after incubation with GSH (10 mM) for 10 min, and were completely dissociated within 3 h (**Figure** **2**A). The gradually decreased hydrodynamic diameter at different time points was in agreement with the TEM observations (Figure 2B). Meanwhile, the solution changed from blue to white, further confirmed the degeneration of ^F127^MOF‐199 NPs (Figure S4, Supporting Information). With the degradation, Cu^2+^ could release to induce copper‐overload‐mediated cuproptosis. Therefore, the released Cu^2+^ was evaluated by ICP‐MS. Figure 2C exhibits a gradual increment of released Cu^2+^, which reached the maximum (67.1%) after 3 h of GSH treatment. The released Cu^2+^ could transform to more toxic Cu^+^ by reacting with GSH or FDX1 to induce cuproptosis.^[^ ^8^ ^]^ To further prove the GSH depletion ability of ^F127^MOF‐199 NPs, 5,5′‐dithiobis‐(2‐nitrobenzoic acid) (DTNB) was used as the probe, as it can react with GSH to produce a yellow product with the absorption at 412 nm.^[^ ^29^ ^]^ Thus, the lower adsorption at 412 nm, the less GSH in the solution. As shown in Figure 2D, the adsorption reduced as the increase of ^F127^MOF‐199 NPs concentration, demonstrating the concentration‐dependent GSH depleting ability of ^F127^MOF‐199 NPs.

The overexpressed endogenous H_2_S is a unique target of CRC, which can enhance cuproptosis by inhibit the copper exporter protein ATP7A.^[^ ^22^ ^]^ To investigate H_2_S‐responsiveness, NaHS was introduced into ^F127^MOF‐199 NPs solution to imitate endogenous H_2_S. Meanwhile, the UV–Vis–NIR absorption spectra were used to monitor the reaction. The color of the solution quickly changed from blue to brownish black (Figure S5, Supporting Information), accompanying with the gradually increased adsorption in NIR‐II region (Figure 2E), implying the generation of copper sulfide with potential applications in NIR‐II PTT. For further investigation, the products were centrifuged and characterized by TEM. As shown in Figure 2F,G, ^F127^MOF‐199 NPs transformed into small fragments with an average size of 16.2 nm. The generated sulfidation product with reduced size is beneficial for faster endocytosis. The EDS elemental mappings revealed the homogenous distribution of Cu and S over the entire NPs (Figure 2H). The XPS and XRD pattern further proved the generation of copper sulfide (Figure 2I and Figure S6, Supporting Information). The spectrum of Cu 2p displayed two pairs doublet peaks, in which the peaks at 932.2 (2p_3/2_) and 952.1 eV (2p_1/2_) were attributed to Cu^+^, while the peaks at 933.6 (2p_3/2_) and 954.1 eV (2p_1/2_) were ascribed to Cu^2+^ (Figure 2I).^[^ ^30^ ^]^ Based on the peak area, the content of Cu^+^ and Cu^2+^ was calculated to be around 70% and 30%, respectively. The binding energies located at 161.8 and 163.2 eV of S 2p spectrum were attributed to 2p_3/2_ and 2p_1/2_ of S^2−^ (Figure 2J).^[^ ^31^ ^]^ Collectively, all these results proved the generation Cu_2−*x*_S NPs by in situ sulfidation.

### 2.3 In Situ Sulfidation Induced PTT and CDT

**Figure 3:**
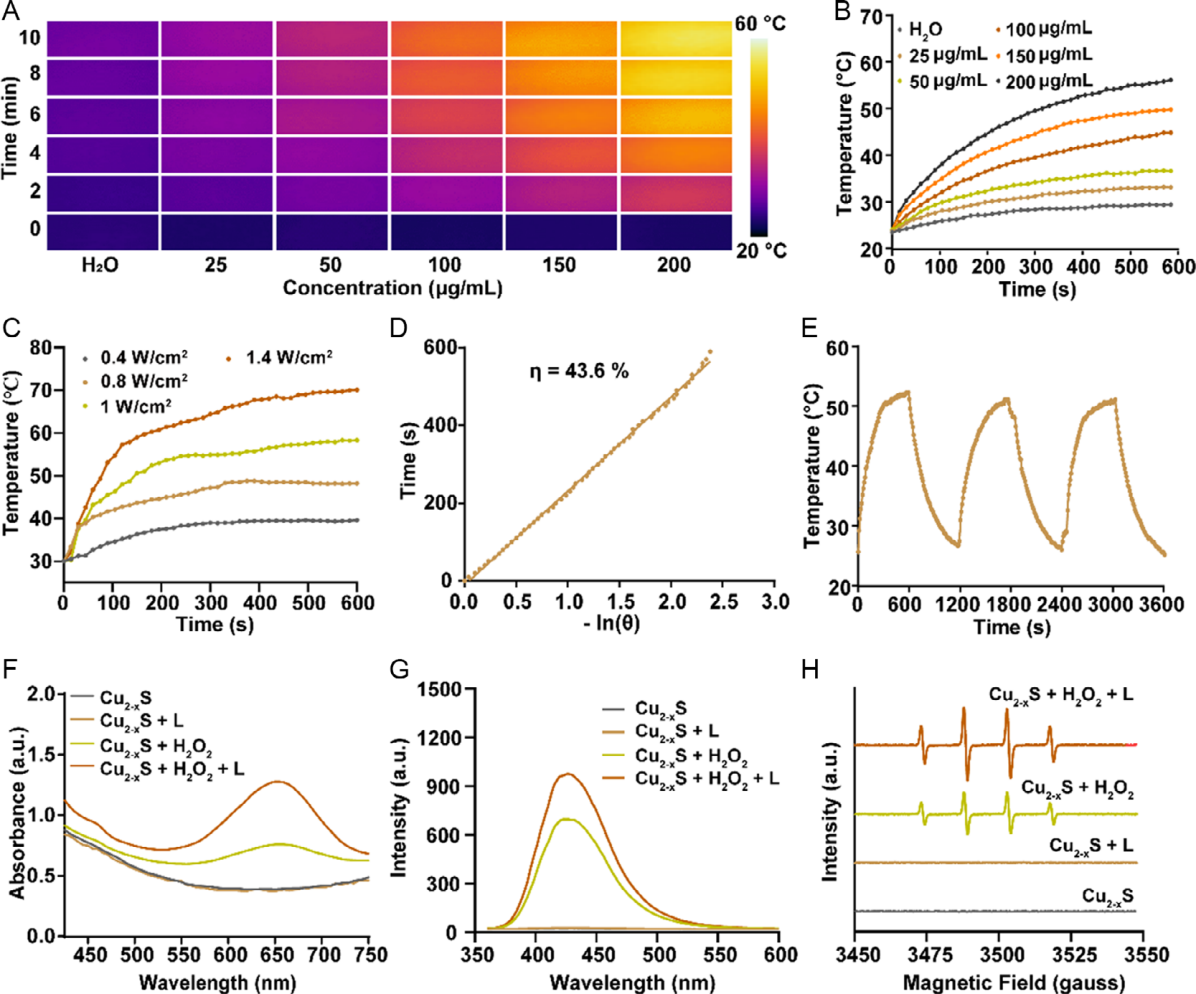
In situ sulfidation‐induced photothermal and chemodynamic performance. A) Photothermal imaging, B) heating curves of Cu_2−*x*_S solution with different concentrations under 1064 nm laser irradiation (1 W cm^−2^, 10 min), and C) heating curves of Cu_2−*x*_S solution (200 μg mL^−1^) under different powers of irridation. D) Linear fitting curve of time versus −In(*θ*) obtained from the cooling period after 10 min of irradiation. E) Photothermal stability analysis of Cu_2−*x*_S solution after three cycles of laser on/off. F) UV–Vis absorption spectra of TMB incubated with Cu_2−*x*_S NPs under different conditions. G) TPA assay of Cu_2−*x*_S NPs under different conditions. H) ESR spectra for detection of •OH under different conditions using DMPO as the trapping agent.

Since the generated Cu_2−*x*_S NPs exhibited strong NIR‐II absorption (Figure 2E), the photothermal performance was investigated under an 1064 nm laser. As illustrated in **Figure** **3**A, the photothermal imaging of pure H_2_O barely changed after 10 min of laser irradiation, while it became much brighter as the increase of Cu_2−*x*_S concentration. The high concentration and power dependence were also observed in the corresponding heating curves (Figure 3B,C). After the laser irradiation (1 W cm^−2^), the temperature of Cu_2−*x*_S solution (200 μg mL^−1^) can reach up to 56.4 °C. The calculated photothermal conversion efficiency was 43.6% (Figure 3D), demonstrating the excellent photothermal conversion ability. Moreover, after three cycles of laser on/off, Cu_2−*x*_S solution still maintain the good photothermal stability (Figure 3E), suggesting the excellent potential in NIR‐II PTT.

It is well‐known that Cu ion with multivalent states can catalyze H_2_O_2_ to produce •OH for CDT.^[^ ^32^ ^]^ Therefore, the ROS generation ability of Cu_2−*x*_S NPs was evaluated by 3,3,5,5‐tetramethylbenzidine (TMB) assay, as TMB can be oxidized by ROS to obtain a blue product with the absorption at ≈650 nm.^[^ ^33^ ^]^ The Cu_2−*x*_S NPs revealed undetectable ROS in absence of H_2_O_2_, as shown by the negligible absorption at 650 nm. However, the absorption at 650 nm remarkably raised for Cu_2−*x*_S NPs plus H_2_O_2_, indicating the generation of ROS by the reaction of Cu_2−*x*_S NPs and H_2_O_2_ (Figure 3F). More importantly, the further increased absorption at 650 nm after laser irradiation suggested the photothermal‐enhanced ROS generation. The terephthalic acid (TPA) assay was then performed to demonstrate that Cu_2−*x*_S NPs could react with H_2_O_2_ to generate •OH. The highest fluorescence signal was detected in Cu_2−*x*_S + H_2_O_2_ + L group (Figure 3G), further confirming the photothermal‐enhanced CDT. As a further proof, the electron spin resonance (ESR) analysis was carried out to detect •OH using 5,5‐dimethyl‐1‐pyrroline‐N‐oxide (DMPO) as the spin‐trapping agent. As shown in Figure 3H, the characteristic 1:2:2:1 signal was observed in Cu_2−*x*_S + H_2_O_2_ group, and the signal was further increased after the laser irradiation, which was consistent with the results of TMB and TPA assays.

### 2.4 In Vitro Therapeutic Efficacy of ^F127^MOF‐199 NPs

**Figure 4:**
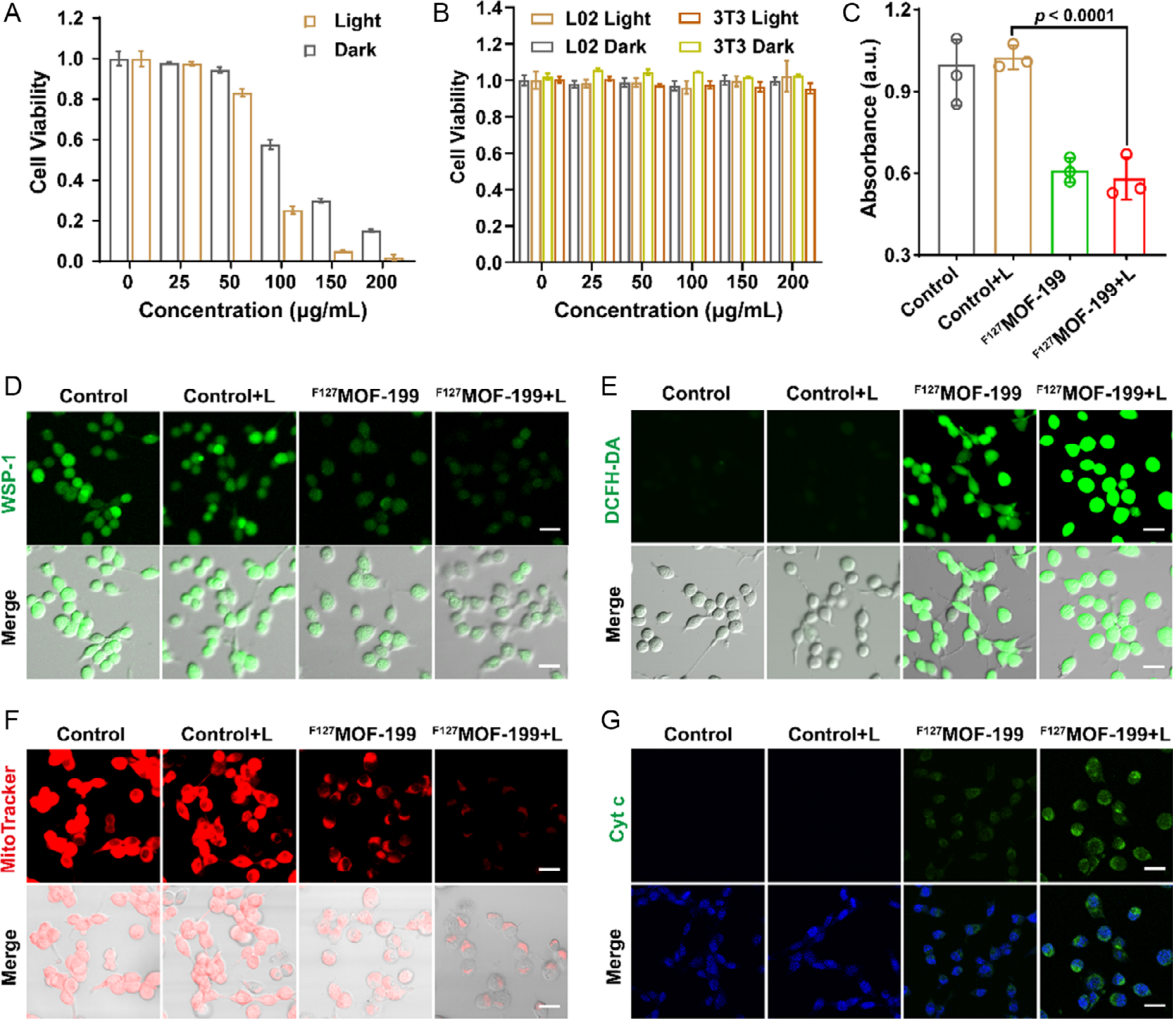
In vitro therapeutic efficacy of ^F127^MOF‐199 NPs. A) The viabilities of ^F127^MOF‐199 NPs against CT26 tumor cells with and without laser irridation. B) The viabilities of ^F127^MOF‐199 NPs against L02 and 3T3 normal cells with and without laser irradiation. C) Intracellular GSH content after different treatments using DTNB as the probe. D) Intracellular H_2_S staining after different treatments using WSP‐1 probe (scale bar = 25 μm). E) Generation of intracellular ROS using DCFH‐DA as the probe (scale bar = 25 μm). F) Distribution of mitochondria by staining with MitoTracker Deep Red FM (scale bar = 25 μm). G) Immunofluorescence staining of cytochrome c (scale bar = 25 μm). Data are presented as the mean ± SD, and statistical analysis was performed using one‐way analysis of variance (ANOVA) and Tukey's tests.

The dual‐responsive behavior and in situ sulfidation‐induced PTT/CDT of ^F127^MOF‐199 NPs motivate us to investigate in vitro therapeutic effect. Firstly, to evaluate the cellular uptake behavior, CT26 colon tumor cells were incubated with Cy5.5‐labeled ^F127^MOF‐199 NPs for different time and analyzed by flow cytometry. The fluorescence was quenched in Cy5.5‐labeled ^F127^MOF‐199 solution due to the close distance between metal center and dye molecule, while the fluorescence recovered after the addition of GSH due to GSH‐induced degeneration of ^F127^MOF‐199 NPs (Figure S7, Supporting Information). The strong intracellular fluorescence detected by flow cytometry further demonstrated the GSH‐responsive decomposition behavior of ^F127^MOF‐199 NPs. Moreover, the mean fluorescence intensity showed that the cellular uptake was greatest after 12 h of incubation (Figure S8, Supporting Information). Subsequently, the cellular cytotoxicity of ^F127^MOF‐199 NPs was evaluated by counting kit‐8 (CCK‐8) assay with both tumor (CT26) and normal (L02 and 3T3) cells. **Figure** **4**A revealed the high toxicity of ^F127^MOF‐199 NPs to CT26 cells under dark condition, while the toxicity was significantly increased after the irradiation of 1064 nm laser, indicating the excellent anticancer effect of synergistic CDT and PTT. In contrast, the toxicity to normal cells (L02 and 3T3) was negligible even at the concentration of 200 μg mL^−1^, suggesting the good biocompatibility of ^F127^MOF‐199 NPs (Figure 4B). The highly selective cytotoxicity to tumor cells rather than normal cells can be attributed to the dual‐responsiveness (GSH and H_2_S) induced CRC targeting of ^F127^MOF‐199 NPs.

To further verify the dual‐responsiveness of ^F127^MOF‐199 NPs in vitro, the consumption of endogenous GSH and H_2_S were examined. The intracellular GSH content was evaluated by analysis of cell lysate supernatant using DTNB as the probe. The lower adsorption at 412 nm indicates the less GSH in solution. The apparently decreased absorption in ^F127^MOF‐199 and ^F127^MOF‐199 + L group compared with the control group verified the GSH consumption ability of ^F127^MOF‐199 NPs (Figure 4C). The intracellular H_2_S was evaluated by using WSP‐1 fluorescence probe. The confocal image clearly displayed the green fluorescence in control group, in accordance with the high expressed H_2_S in CT26 cells. However, the fluorescence intensity was evidently decreased in ^F127^MOF‐199 and ^F127^MOF‐199 + L group, indicating the consumption of intracellular H_2_S by ^F127^MOF‐199 NPs (Figure 4D). This result was in consistent with the in situ sulfidation of ^F127^MOF‐199 NPs, where the intracellular H_2_S was consumed during the reaction.

The good in vitro therapeutic effect of ^F127^MOF‐199 NPs motivates us to further explore the therapeutic mechanism. Since the generated Cu_2−*x*_S NPs by in situ sulfidation have shown great potential in PTT/CDT, the intracellular ROS level was firstly evaluated by 2,7‐dichlorodihydrofluorescein diacetate (DCFH‐DA) fluorescence probe. As shown in Figure 4E, the obvious fluorescence enhancement is observed in ^F127^MOF‐199 group, indicating the excellent ROS production ability. The further increased fluorescence intensity in ^F127^MOF‐199 + L group can be ascribed to the photothermal‐enhanced CDT. These results are in good agreement with the TMB assay hereinbefore. Since the excessive intracellular ROS is usually accompanied with mitochondrial dysfunction,^[^ ^34^ ^]^ the intracellular mitochondria distribution was analyzed by MitoTracker Deep Red FM, which can specially stain mitochondria with red fluorescence. As expected, the red fluorescence clearly decreased in presence of ^F127^MOF‐199 NPs, and the least fluorescence intensity was observed in ^F127^MOF‐199 + L group, implying most of the mitochondria are damaged (Figure 4F). Cytochrome c, anchored in the mitochondrial intermembrane space, can release into the cytoplasm when mitochondrial membrane is damaged.^[^ ^35^ ^]^ Therefore, the level of cytochrome c in the cytoplasm was accessed by immunofluorescence. The highest fluorescence intensity can be observed in ^F127^MOF‐199 + L group (Figure 4G), indicating the efficient release of cytochrome c into cytoplasm.

### 2.5 Synergistic Cuproptosis/Pyroptosis and Induced In Vitro Immune Response

**Figure 5:**
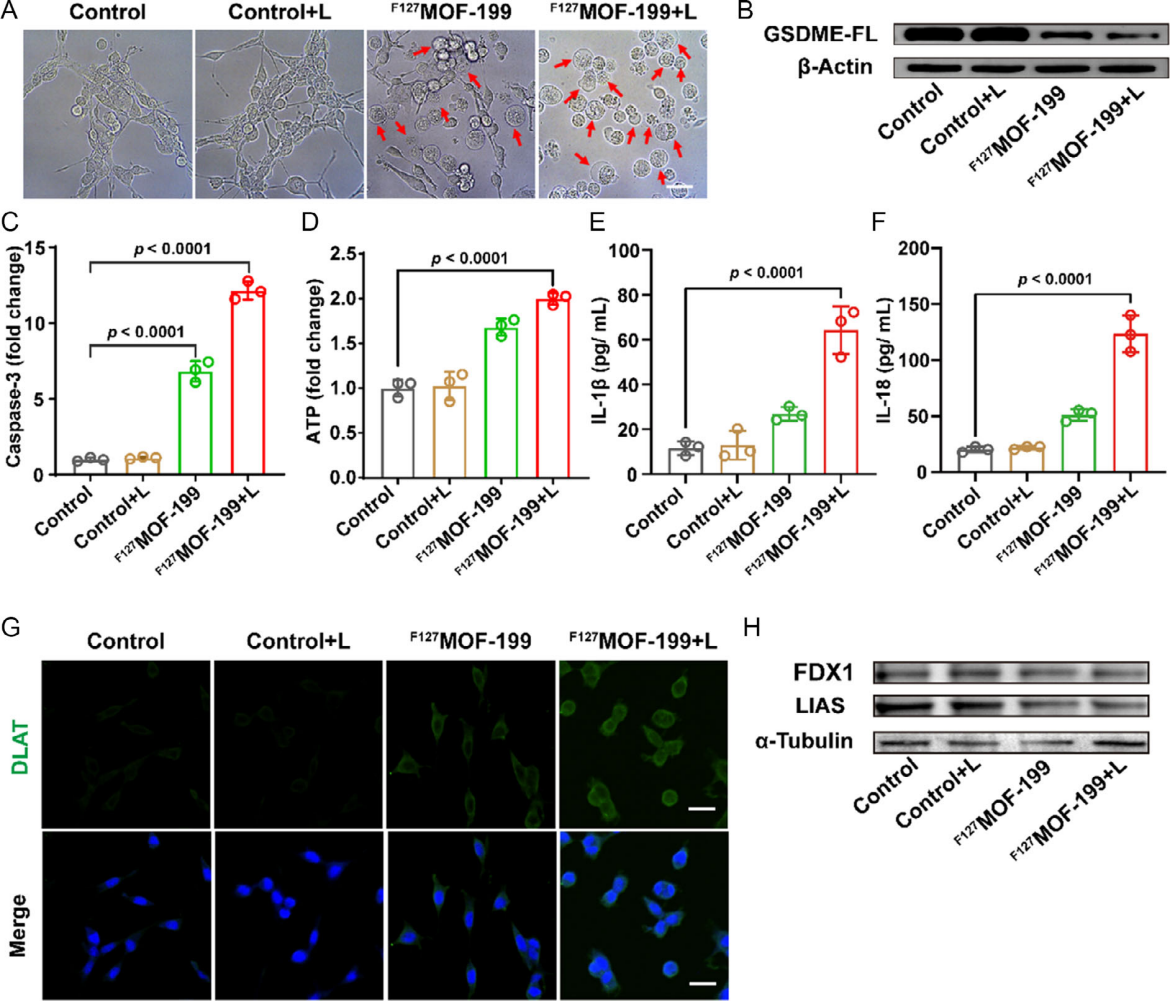
^F127^MOF‐199 NPs induced cell death. A) Morphology of CT26 cells after different treatments (scale bar = 25 μm). The red arrows indicate the pyroptosis cells. B) Western blotting analysis of full‐length GSDME (GSDME‐FL) in CT26 cells after different treatments. C) The fluorescence quantification of intracellular caspase‐3 activation by flow cytometry analysis. D) The released ATP after different treatments. E,F) The release of IL‐1β and IL‐18 in cell supernatant by ELISA. G) DLAT immunofluorescence imaging after different treatments (scale bar = 25 μm). H) Western blotting analysis of cuproptosis‐related proteins, FDX1, and LIAS. Data are presented as the mean ± SD, and statistical analysis was performed using one‐way ANOVA and Tukey's tests.

Although the release of cytochrome c is usually related to apoptosis, Shao recently reported that GSDME can convert apoptosis to pyroptosis in the cells with high GSDME expression.^[16a]^ The high selective cytotoxicity to CT26 with high expressed GSDME motivates us to investigate ^F127^MOF‐199 NPs‐induced cell death. Since pyroptosis is characterized by membrane perforation and cell swelling, the inverted microscopy was used to detect the cell morphology after different treatments. Compared with the normal cell morphology in control or control+L group, a large number of cells displayed swelling and ballooning (red arrows) in the groups containing ^F127^MOF‐199, suggesting the occurrence of pyroptosis (**Figure** **5**A and Figure S9A, Supporting Information). The increased proportion of pyroptotic cells under laser irradiation implied PTT‐enhanced cell pyroptosis (Figure S9B, Supporting Information). Consistent with this, a 10.7‐fold increase of intracellular activated caspase‐3 was observed in ^F127^MOF‐199 + L group relative to control group (Figure S10, Supporting Information and Figure 5C). Caspase‐3 is considered as a pyroptosis inducer which cleaves full‐length GSDME (GSDME‐FL) into GSDME‐N terminal, thus the reduced GSDME‐FL is the critical feature of pyroptosis. As expected, the western blot displayed the apparently reduced GSDME‐FL in ^F127^MOF‐199 + L (Figure 5B). What is more, the apparently increased intracellular ATP level further proved ^F127^MOF‐199 NPs‐induced pyroptosis (Figure 5D). Moreover, the contents of proinflammatory cytokines released by pyroptosis were evaluated. As shown in Figure 5E,F, 5.6‐fold elevated level of IL‐1β and 6.1‐fold elevated level of IL‐18 were observed in cell supernatant in ^F127^MOF‐199 + L group compared to control group. Collectively, the occurrence of pyroptosis in ^F127^MOF‐199 NPs was demonstrated at the cellular level.

Inspired by GSH‐responsive release of Cu^2+^ for ^F127^MOF‐199 NPs, cell cuproptosis was investigated. Cuproptosis occurs by means of direct binding of copper to DLAT, which causes DLAT aggregation and subsequent proteotoxic stress and cell death.^[^ ^8^ ^]^ DLAT oligomerization was studied by immunofluorescence imaging and western blot assay. As shown in Figure 5G, the cells treated with control and control+L groups displayed negligible DLAT foci, while the MOF‐treated groups exhibited pronounced DLAT foci, especially ^F127^MOF‐199 + L group. The foci formation was attributed to the aberrant oligomerization of DLAT, which was one of the characteristics of cuproptosis. Consistent with this, the obvious DLAT oligomers were observed in ^F127^MOF‐199 and ^F127^MOF‐199 + L group, while the oligomers were negligible in control and control+L group (Figure S11, Supporting Information). Moreover, the decreased levels of Fe‐S cluster protein (FDX1) and lipoyl synthase (LIAS), the hallmarks of cuproptosis, were observed in ^F127^MOF‐199 and ^F127^MOF‐199 + L‐treated cells by western blot assay (Figure 5H). To further confirm that cuproptosis plays an important role in ^F127^MOF‐199 NPs‐induced cell death, the effects of cuproptosis inhibitor (Rotenone) on the cytotoxicity of ^F127^MOF‐199 were evaluated in CT26 cancer cells. The cytotoxicity was measured after the treatments of ^F127^MOF‐199 + L with and without Rotenone (0.1 μM), respectively. The viability was expressed as the fold change of CT26 cells treated with ^F127^MOF‐199 + L and Rotenone relative to that of ^F127^MOF‐199 + L without Rotenone. As shown in Figure S12 (Supporting Information), the apparently increased viability after the combined treatment of ^F127^MOF‐199 NPs and Rotenone indicated the important role of cuproptosis in ^F127^MOF‐199 NPs‐induced cell death. All these results indicated the occurrence of cuproptosis in ^F127^MOF‐199 NPs‐induced cytotoxicity.

**Figure 6:**
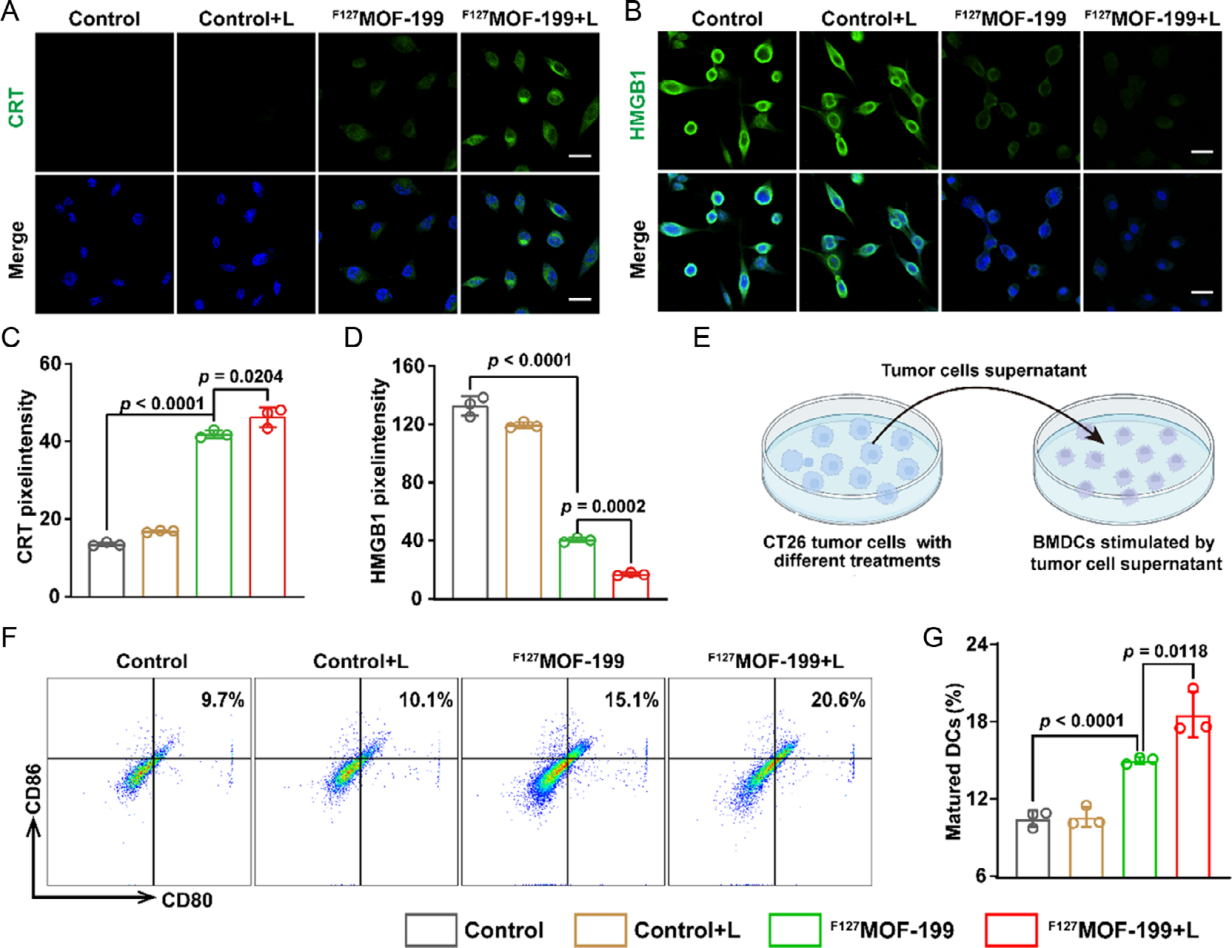
^F127^MOF‐199 NPs‐induced ICD and immune response. A) CRT and B) HMGB1 immunofluorescence imaging after different treatments (scale bar = 100 μm). C,D) Relative fluorescence intensity of CRT (C) and HMGB1 (D). E) Schematic illustration of BMDCs immune stimulation in vitro. F) Representative flow cytometry analysis and G) corresponding quantification of mature DCs after stimulated by CT26 cell supernatant with different treatments. Data are presented as the mean ± SD, and statistical analysis was performed using one‐way ANOVA and Tukey's tests.

The above results have demonstrated ^F127^MOF‐199 NPs can synergistically trigger pyroptosis and cuproptosis. To further verify the in vitro immune response induced by ^F127^MOF‐199 NPs, the typical markers of ICD, CRT, and HMGB1, were analyzed. **Figure** **6**A,B showed the immunofluorescence imaging of CRT and HMGB1 of CT26 cells under different treatments, where the highest CRT exposure and HMGB1 release can be observed in ^F127^MOF‐199 + L group. The mean fluorescence intensity analysis revealed a 4.1‐fold increase in CRT exposure (Figure 6C) and an 8.2‐fold increase in HMGB1 release (Figure 6D) for ^F127^MOF‐199 + L group compared with control group. The efficient ICD can activate dendritic cells (DCs), enhance antigen presentation, and convert “cold tumor” to “hot tumor”.^[^ ^36^ ^]^ In order to investigate the activation of DCs by ^F127^MOF‐199 NPs, bone‐marrow‐derived dendritic cells (BMDCs) were separated from C57BL6 mice and incubated with CT26 cells supernatant after different treatments (Figure 6E). The percentages of DCs maturation in vitro were analyzed by detecting unregulated levels of costimulatory molecules of CD80 and CD86. As shown in Figure 6F,G, control and control+L groups hardly induced DC maturation after 12 h of incubation. In contrast, the matured DCs increased to 20.6% in ^F127^MOF‐199 + L group, which was much higher than 9.7% in control group. A comprehensive look at the aforementioned results, we confirmed that ^F127^MOF‐199 NPs can induce ICD to efficiently activate DCs, which possibly facilitate in vivo immune response.

### 2.6 In Vivo Therapeutic Efficiency of ^F127^MOF‐199 NPs

**Figure 7:**
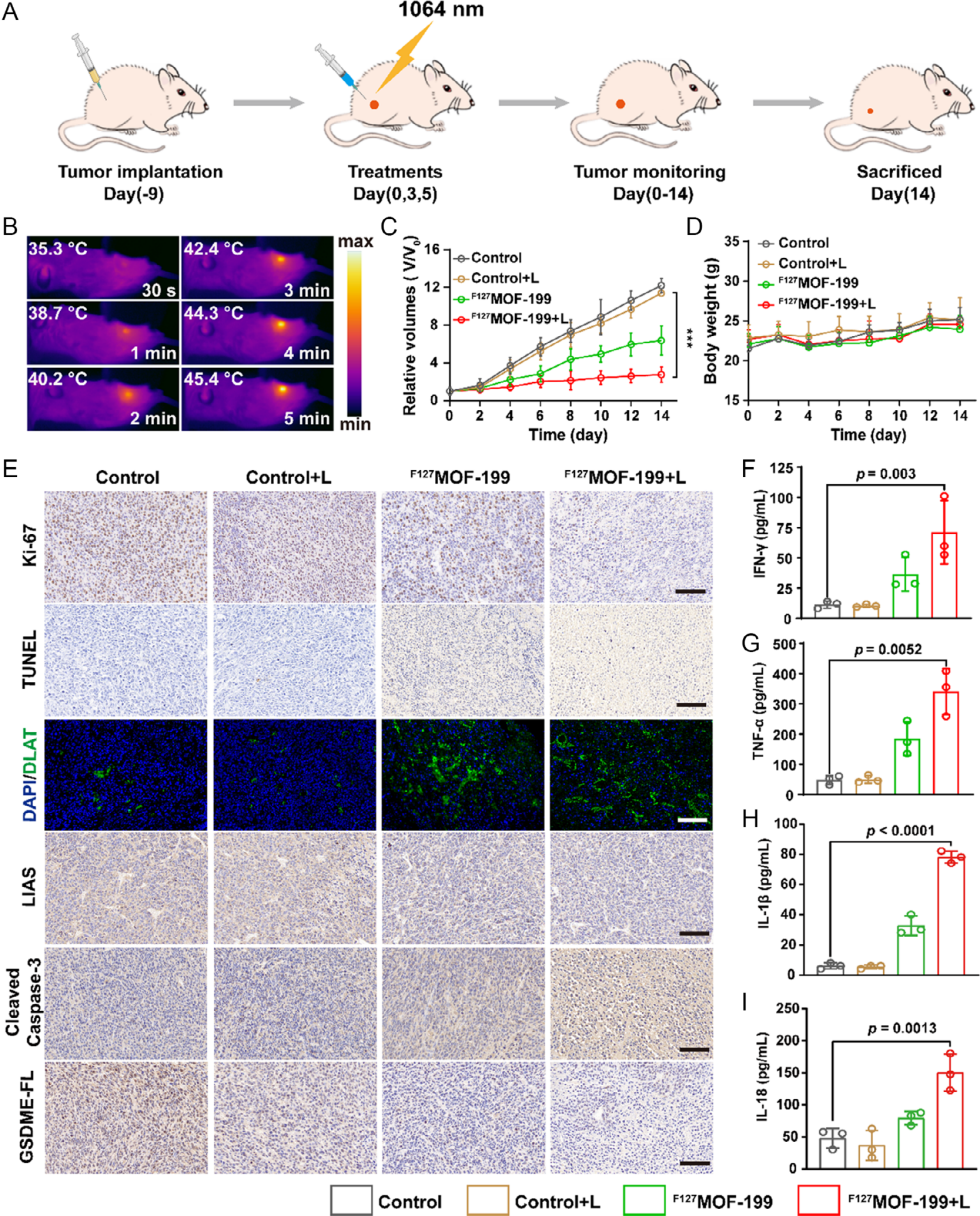
In vivo antitumor efficacy of ^F127^MOF‐199 NPs against CRC model. A) Time schedule of the treatment. B) Photothermal imaging of BALB/c mice bearing CT26 tumors. C) Tumor growth curve. D) Body weight curve. E) Ki‐67, TUNNEL, DLAT, LIAS, cleaved caspase‐3, GSDME‐FL staining of tumor sections (scale bar = 100 μm). F−I) The released IFN‐γ, TNF‐α, IL‐1β, and IL‐18 in mice serum detected by ELISA after different treatments. Data are presented as the mean ± SD, and statistical analysis was performed using one‐way ANOVA and Tukey's tests.

Encouraged by the excellent in vitro antitumor effect and immune activation by ^F127^MOF‐199 NPs, the in vivo therapeutic efficacy was investigated by H_2_S‐overexpressed CT26‐tumor‐bearing mice. After tumors grew to ≈150 mm^3^, the mice were randomly divided into four groups, and treated with control, control+L, ^F127^MOF‐199, and ^F127^MOF‐199 + L, respectively. In order to determine the optimal time for precise PTT, the intratumoral retention behavior was evaluated by Cy5.5‐labeled ^F127^MOF‐199 NPs. The fluorescence intensity in the tumor region gradually increased and eventually reached the maximum at 12 h (Figure S13, Supporting Information), implying the decomposition and in situ sulfidation of ^F127^MOF‐199 NPs. Therefore, PTT was performed by irradiation with 1064 nm laser at 12 h postinjection of NPs (**Figure** **7**A). As shown in Figure 7B, the tumor temperature of ^F127^MOF‐199 + L group reached up to 45.4 °C after receiving the irradiation of 1 W cm^−2^ for 5 min, demonstrating the efficient PTT, which is beneficial for pyroptosis activation. The tumor growth curve of control+L group increased rapidly similar to control group, indicating the irradiation alone can hardly inhibit tumor growth. In contrast, the tumor volumes and weights were greatly inhibited in ^F127^MOF‐199 NPs+L group (Figure 7C and Figure S14A, Supporting Information), manifesting the excellent therapeutic efficiency of synergistic cuproptosis and pytoptosis. Moreover, the prolonged survival test displayed much higher survival rate of ^F127^MOF‐199 NPs+L group (Figure S14B, Supporting Information), further proved the therapeutic efficiency of ^F127^MOF‐199 NPs plus irradiation. The biosafety of ^F127^MOF‐199 NPs was validated by the examination of mice weight, blood tests, and hematoxylin and eosin (H&E) staining images of major organs. The negligible change in body weights (Figure 7D), blood parameters (Figure S15, Supporting Information), and H&E analysis of major organs (Figure S16, Supporting Information) demonstrated the excellent biosafety of ^F127^MOF‐199 NPs for in vivo therapy.

The antitumor effect was further evaluated by the sections of tumor tissue. The H&E staining images showed the most intensive tumor cell damage in ^F127^MOF‐199 + L group, while no obvious influence in control group (Figure S17, Supporting Information). The immunohistochemical staining of Ki‐67 displayed the dramatically suppression of tumor cell proliferation in ^F127^MOF‐199 + L group, which was consistent with the TUNEL staining results exhibiting the most severe apoptosis/necrosis in ^F127^MOF‐199 + L group (Figure 7E). To explore the antitumor mechanism in vivo, the cuproptosis and pyroptosis related proteins were evaluated in a tumor tissue. As revealed in Figure 7E, the most severe DLAT aggregation and LIAS loss, the most pronounced caspase‐3 cleavage, and most significant GSDME‐FL downregulation were observed in ^F127^MOF‐199 + L group, manifesting the occurrence of cuproptosis and pyroptosis in vivo. The synergistic cuproptosis and pyroptosis could induce the release of inflammatory factors. The enzyme‐linked immune sorbent assay (ELISA) data indicated that ^F127^MOF‐199 + L can boost the release of interleukin‐1β (IL‐1β), interleukin‐18 (IL‐18), tumor necrosis factor‐α (TNF‐α), and interferon‐γ (IFN‐γ), resulting in the acute inflammatory responses to activate immune cells (Figure 7F–I).

**Figure 8:**
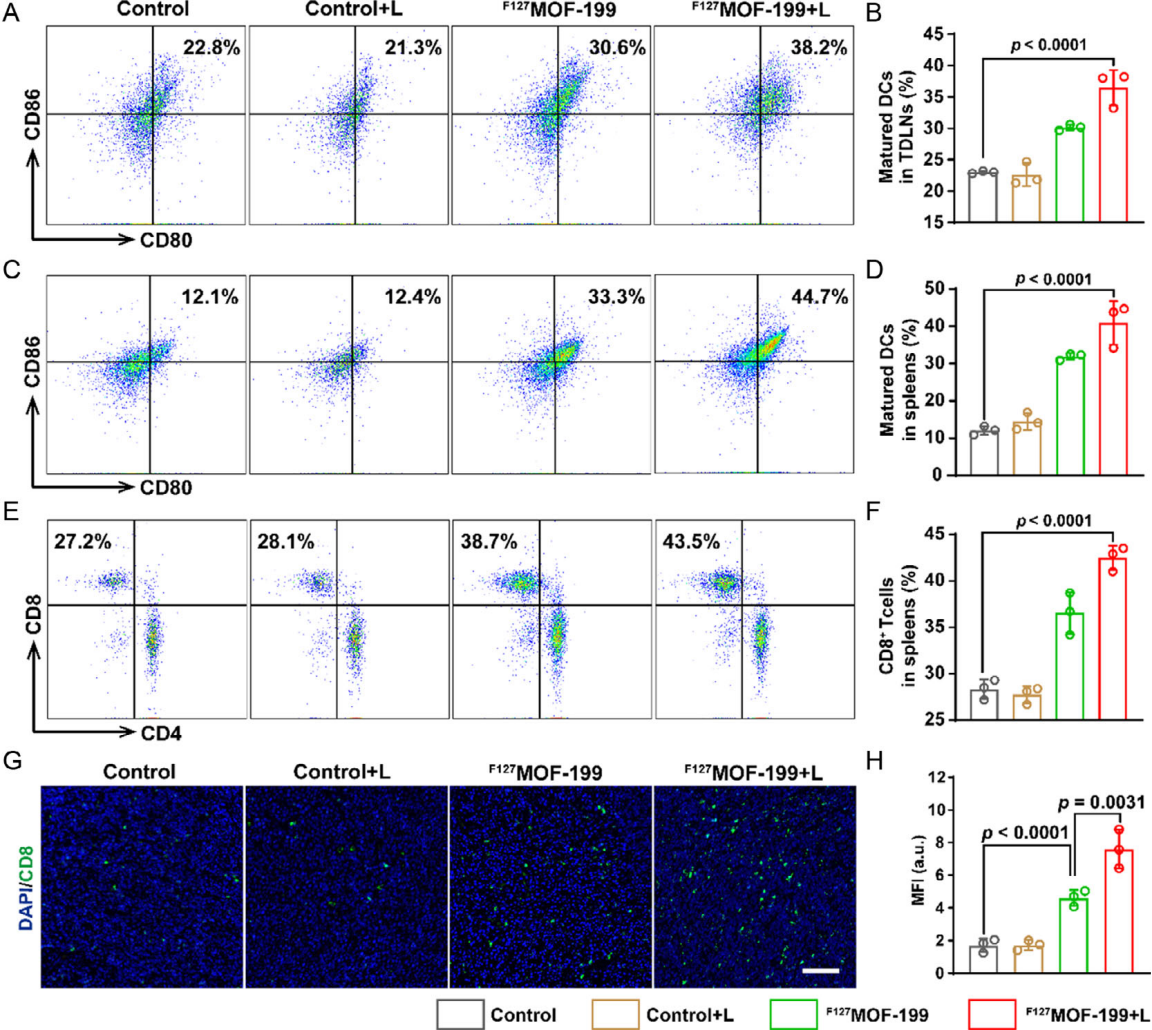
In vivo immune activation effects in CRC model. A) Representative flow cytometry data and B) quantification of matured DCs in TDLNs of CT26‐tumor‐bearing mice after different treatments. C) Representative flow cytometry data and D) quantification of matured DCs in spleens of CT26‐tumor‐bearing mice after different treatments. E) Representative flow cytometry data and F) quantification of CD8^+^ T‐cells in spleen of CT26‐tumor‐bearing mice after different treatments. G,H) Images of immunofluorescence staining and the corresponding fluorescence quantification of CD8^+^ T‐cells in tumor sections (scale bar = 100 μm). Data are presented as the mean ± SD, and statistical analysis was performed using one‐way ANOVA and Tukey's tests.

To uncover the antitumor immunity effect in vivo, the mice were sacrificed on the 15^th^ day, and the immune cells in tumor draining lymph nodes (TDLNs), spleens, and cytokines in serum were acquired for immune analysis. The single cell suspensions of the TDLNs and spleens were stained with fluorochrome‐conjugated anti‐mouse antibodies for flow cytometric analysis. The mature DCs in TDLNs were firstly assessed. As shown in **Figure** **8**A,B, the percentage of mature DCs in ^F127^MOF‐199 + L group (38.2%) reached up to 1.7 folds of the control group (22.8%). Then, the significantly elevated mature DCs (^F127^MOF‐199 + L vs. control = 44.7% vs. 12.1%) was found in spleens (Figure 8C,D), which could potentially activate T‐cells. As expected, the splenic‐activated CD8^+^ T‐cells increased by 1.6 folds, which is 27.2% in the control group and 43.5% in ^F127^MOF‐199 + L group (Figure 8E,F). Meanwhile, the fluorochrome‐conjugated tumor slides also revealed the highest T‐cell infiltration in ^F127^MOF‐199 + L group (Figure 8G,H). Altogether, these results demonstrated that ^F127^MOF‐199 NPs can induce cuproptosis and pyroptosis to activate immunity for tumor therapy.

## 3 Conclusions

We have successfully fabricated GSH/H_2_S dual‐responsive ^F127^MOF‐199 NPs for tumor‐targeted immunotherapy by synergistic cuproptosis and pyroptosis. The ^F127^MOF‐199 NPs exhibited GSH‐responsive degeneration behavior and H_2_S‐activated in situ sulfidation. Upon cellular internalization, ^F127^MOF‐199 NPs dissociated rapidly to release Cu^2+^, which converted to more toxic Cu^+^, and eventually induce copper‐overload‐mediated cuproptosis. In the meantime, the in situ sulfidation successfully transformed ^F127^MOF‐199 NPs to Cu_2−*x*_S NPs, avoiding the phototoxicity to normal cells. The activation of PTT/CDT for Cu_2−*x*_S NPs promoted the release of cytochrome c followed by the activation of caspase‐3, leading to the cleave of GSDME and realizing of pyroptosis. Combined with H_2_S‐activated pyroptosis and cuproptosis, ICD was successfully induced, which efficiently promoted the systemic immune response both in vitro and in vivo. The CRC mouse model was chosen to verify the therapeutic effect of ^F127^MOF‐199 NPs, which exhibited the excellent antitumor immunity activity by enhanced DCs maturation, CT8^+^ T‐cell activation, and apparently tumor growth inhibition. This work presents a simple strategy to realize tumor‐targeted immunotherapy of CRC by GSH/H_2_S dual‐responsive activation of cuproptosis and pyroptosis.

## Conflict of Interest

The authors declare no conflict of interest.

## Supporting information

Supplementary Material

## Data Availability

The data that support the findings of this study are available from the corresponding author upon reasonable request.

## References

[1] F. Bray , J. Ferlay , I. Soerjomataram , R. L. Siegel , L. A. Torre , A. Jemal , CA Cancer J. Clin. 2018, 68, 394. 30207593 10.3322/caac.21492

[2] H. Yin , T. Gao , J. Xie , Z. Huang , X. Zhang , F. Yang , W. Qi , Z. Yang , T. Zhou , G. Gao , X. Yang , Mol. Oncol. 2021, 15, 3490. 34288405 10.1002/1878-0261.13064 PMC8637553

[3] a) C. G. Drake , E. J. Lipson , J. R. Brahmer , Nat. Rev. Clin. Oncol. 2014, 11, 24; 24247168 10.1038/nrclinonc.2013.208 PMC4086654

[4] a) J. Galon , A. Costes , F. Sanchez-Cabo , A. Kirilovsky , B. Mlecnik , C. Lagorce-Pages , M. Tosolini , M. Camus , A. Berger , P. Wind , F. Zinzindohoue , P. Bruneval , P. H. Cugnenc , Z. Trajanoski , W. H. Fridman , F. Pages , Science 2006, 313, 1960; 17008531 10.1126/science.1129139

[5] a) G. Kroemer , L. Galluzzi , O. Kepp , L. Zitvogel , Annu. Rev. Immunol. 2013, 31, 51; 23157435 10.1146/annurev-immunol-032712-100008

[6] a) Z. Li , X. Lai , S. Fu , L. Ren , H. Cai , H. Zhang , Z. Gu , X. Ma , K. Luo , Adv. Sci. 2022, 9, 2201734; 10.1002/advs.202201734 PMC9353475 35652198

[7] a) S. Fulda , Int. J. Cancer 2009, 124, 511; 19003982 10.1002/ijc.24064

[8] P. Tsvetkov , S. Coy , B. Petrova , M. Dreishpoon , A. Verma , M. Abdusamad , J. Rossen , L. Joesch-Cohen , R. Humeidi , R. D. Spangler , J. K. Eaton , E. Frenkel , M. Kocak , S. M. Corsello , S. Lutsenko , N. Kanarek , S. Santagata , T. R. Golub , Science 2022, 375, 1254. 35298263 10.1126/science.abf0529 PMC9273333

[9] a) T. Tsang , C. I. Davis , D. C. Brady , Curr. Biol. 2021, 31, R421; 33974864 10.1016/j.cub.2021.03.054

[10] a) M. A. Kahlson , S. J. Dixon , Science 2022, 375, 1231; 35298241 10.1126/science.abo3959

[11] a) W. J. Xu , J. M. Qian , G. H. Hou , T. B. Wang , J. L. Wang , Y. P. Wang , L. J. Yang , X. K. Cui , A. L. Suo , Adv. Funct. Mater. 2022, 32, 2205013;

[12] B. Guo , F. Yang , L. Zhang , Q. Zhao , W. Wang , L. Yin , D. Chen , M. Wang , S. Han , H. Xiao , N. Xing , Adv. Mater. 2023, 35, 2212267. 10.1002/adma.202212267 36916030

[13] Q. Xiang , C. Yang , Y. Luo , F. Liu , J. Zheng , W. Liu , H. Ran , Y. Sun , J. Ren , Z. Wang , Small 2022, 18, 2107809. 10.1002/smll.202107809 35143709

[14] a) J. Ding , K. Wang , W. Liu , Y. She , Q. Sun , J. Shi , H. Sun , D. C. Wang , F. Shao , Nature 2016, 535, 111; 27281216 10.1038/nature18590

[15] a) W. Gao , X. Wang , Y. Zhou , X. Wang , Y. Yu , Signal Transduct. Target. Ther. 2022, 7, 196; 35725836 10.1038/s41392-022-01046-3 PMC9208265

[16] a) Y. Wang , W. Gao , X. Shi , J. Ding , W. Liu , H. He , K. Wang , F. Shao , Nature 2017, 547, 99; 28459430 10.1038/nature22393

[17] a) J. Zhang , Y. Lin , Z. Lin , Q. Wei , J. Qian , R. Ruan , X. Jiang , L. Hou , J. Song , J. Ding , H. Yang , Adv. Sci. 2022, 9, 2103444; 10.1002/advs.202103444 PMC8844476 34927373

[18] a) S. M. Dong , Y. S. Dong , T. Jia , S. K. Liu , J. Liu , D. Yang , F. He , S. L. Gai , P. P. Yang , J. Lin , Adv. Mater. 2020, 32, 2002439; 10.1002/adma.202002439 32914495

[19] D. Tang , X. Chen , G. Kroemer , Cell Res. 2022, 32, 417. 35354936 10.1038/s41422-022-00653-7 PMC9061796

[20] J. Wan , X. Zhang , Z. Li , F. Mo , D. Tang , H. Xiao , J. Wang , G. Rong , T. Liu , Adv. Healthcare Mater. 2023, 12, 2202710. 10.1002/adhm.202202710 36527737

[21] a) C. Szabo , C. Coletta , C. Chao , K. Modis , B. Szczesny , A. Papapetropoulos , M. R. Hellmich , Proc. Natl. Acad. Sci. USA 2013, 110, 12474; 23836652 10.1073/pnas.1306241110 PMC3725060

[22] N. Goto , H. Hara , M. Kondo , N. Yasuda , T. Kamiya , K. Okuda , T. Adachi , Metallomics 2020, 12, 868. 32315022 10.1039/d0mt00015a

[23] a) L. An , X. Wang , X. Rui , J. Lin , H. Yang , Q. Tian , C. Tao , S. Yang , Angew. Chem., Int. Ed. 2018, 57, 15782; 10.1002/anie.201810082 30307092

[24] a) K. Wang , Q. Sun , X. Zhong , M. Zeng , H. Zeng , X. Shi , Z. Li , Y. Wang , Q. Zhao , F. Shao , J. Ding , Cell 2020, 180, 941; 32109412 10.1016/j.cell.2020.02.002

[25] a) Y. Wang , W. Wu , J. Liu , P. N. Manghnani , F. Hu , D. Ma , C. Teh , B. Wang , B. Liu , ACS Nano 2019, 13, 6879; 31194910 10.1021/acsnano.9b01665

[26] Q. Liu , L.-N. Jin , W.-Y. Sun , Chem. Commun. 2012, 48, 8814. 10.1039/c2cc34192a 22836446

[27] J. Shen , W. Zhou , M. Jia , X. Yang , J. Lin , L. An , Q. Tian , S. Yang , ACS Appl. Bio Mater. 2021, 4, 5753. 10.1021/acsabm.1c00521 35006738

[28] B. Ma , S. Wang , F. Liu , S. Zhang , J. Duan , Z. Li , Y. Kong , Y. Sang , H. Liu , W. Bu , L. Li , J. Am. Chem. Soc. 2019, 141, 849. 30541274 10.1021/jacs.8b08714

[29] a) X. Meng , D. Li , L. Chen , H. He , Q. Wang , C. Hong , J. He , X. Gao , Y. Yang , B. Jiang , G. Nie , X. Yan , L. Gao , K. Fan , ACS Nano 2021, 15, 5735; 33705663 10.1021/acsnano.1c01248

[30] W. Qin , J. Z. Huang , C. S. Yang , Q. Yue , S. Z. Chen , M. D. Wang , S. B. Gao , X. Zhou , X. L. Yang , Y. Zhang , Adv. Funct. Mater. 2023, 33, 2209748.

[31] Z. Yang , Y. Luo , Y. Hu , K. Liang , G. He , Q. Chen , Q. Wang , H. Chen , Adv. Funct. Mater. 2021, 31, 2007991.

[32] X. Zhong , X. Wang , L. Cheng , Y. A. Tang , G. Zhan , F. Gong , R. Zhang , J. Hu , Z. Liu , X. Yang , Adv. Funct. Mater. 2020, 30, 1907954.

[33] S. Li , L. Shang , B. Xu , S. Wang , K. Gu , Q. Wu , Y. Sun , Q. Zhang , H. Yang , F. Zhang , L. Gu , T. Zhang , H. Liu , Angew. Chem., Int. Ed. 2019, 58, 12624. 10.1002/anie.201904751 31237404

[34] W. Zhang , X. Hu , Q. Shen , D. Xing , Nat. Commun. 2019, 10, 1704. 30979885 10.1038/s41467-019-09566-3 PMC6461692

[35] a) C. Garrido , L. Galluzzi , M. Brunet , P. E. Puig , C. Didelot , G. Kroemer , Cell Death Differ. 2006, 13, 1423; 16676004 10.1038/sj.cdd.4401950

[36] Y. Li , X. Liu , X. Zhang , W. Pan , N. Li , B. Tang , Chem. Commun. 2021, 57, 12087. 10.1039/d1cc04604g 34714302

