# Supplementary material for: High Immunogenic Cuproptosis Evoked by In Situ Sulfidation‐Activated Pyroptosis for Tumor‐Targeted Immunotherapy of Colorectal Cancer
Source: Small Sci. 2024 Jan 17;4(3):2300164. doi: 10.1002/smsc.202300164 (PMC11935246; doi:10.1002/smsc.202300164)
Supplement: Supplementary file 1 — Supplementary Material [file SMSC-4-2300164-s001.pdf]

High Immunogenic Cuproptosis Evoked by In Situ Sulfidation-Activated Pyroptosis for Tumor-Targeted Immunotherapy of Colorectal Cancer

Wentao Xiao, Kuiming Qu, Wei Zhang, Lunhui Lai, Lei He, Fang Cheng, Lianhui Wang**

State Key Laboratory for Organic Electronics and Information Displays & Jiangsu Key Laboratory for Biosensors, Institute of Advanced Materials (IAM), Nanjing University of Posts and Telecommunications, Nanjing 210023, P. R. China.

E-mail: iamfcheng@njupt.edu.cn, iamlhwang@njupt.edu.cn

Experimental Section

Chemicals

$Cu(NO_3)_2 \cdot 3H_2O$ (98%) and H_2O_2 (30%) were purchased from Sinopharm. Triethylamine (TEA, 98%), benzene-1,3,5-tricarboxylate (H_3btc, 98%), pluronic F127, Glutathione (GSH, 98%), 3,3',5,5'-Tetramethylbenzidine (TMB, 98%) were purchased from Aladdin. Sodium hydrosulfide hydrate ($NaHS \cdot xH_2O$) was purchased from Sigma. ROS Assay Kit, Mito-Tracker Red CMXRos, Cell Counting Kit-8 (CCK-8), GreenNuc™ Live Cell Caspase-3 Activity Assay Kit, Glutathione reductase assay kit (DTNB method), cytochrome c antibody and ATP assay Kit were purchased from Beyotime. Anti-HMGB1 and Anti-CRT were purchased from Servicebio. PerCP/Cyanine5.5 anti-mouse CD4, APC anti-mouse CD3, FITC anti-mouse CD4, PE anti-mouse CD8a, PE/Cyanine7 anti-mouse CD11c, APC anti-mouse CD80, PE anti-mouse CD86 and TruStain FcX™ (anti-mouse CD16/32) were purchased from Biolegend. TNF alpha Mouse uncoated ELISA Kit and IFN gamma Mouse uncoated ELISA Kit were purchased from Invitrogen. All chemicals were used as received without further purification, and deionized (DI) water (18.2 MΩ·cm-1) was used for solution preparation.

Characterizations

The transmission electron microscopy (TEM) images were taken using a Hitachi HT7700 microscope operated at 100 kV. The high-angle annular dark-field scanning TEM (HAADF-STEM) images was acquired with a FEI Talos F200X microscope operated at 200 kV. The scanning electron microscopy (SEM) images were taken using a Hitachi S-4800 microscope. The size distribution was measured by a dynamic light scattering device (ZetaPALS). The X-ray diffraction (XRD) patterns were tested with a Single Crystal X-ray Diffractometer (Bruker D8 Quest). Photothermal imaging were carried out by thermal imagery (FOTRIC 225). UV-vis-NIR extinction spectra were recorded using a spectrophotometer (Shimadzu UV-3600). Confocal images were acquired by confocal laser scanning microscope (OLYMPUS, FV1000MPE). The inductively coupled plasma mass spectrometry (ICP-MS) were recorded using a Nexion 2000 ICP-MS instrument (PerkinElmer). CCK-8 kit was conducted using a Microplate reader (SpectraMax M2). For flow cytometry analysis, the samples were examined by flow cytometry (NovoCyte) and data were processed using FlowJo software. The mice were imaged by small animal fluorescence imaging system (IVIS LUMINA K).

Synthesis of ^{F127}MOF-199 NPs

A two-step procedure was used to synthesis MOF-199 NPs.[1] Firstly, H_3btc (2.1 g, 0.01 mol) was dissolved in TEA (10 mL, 30 wt% aqueous solution), and dried under vacuum to get triethylamine salt of benzene-1,3,5-tricarboxylic acid (btc). Then, $Cu(NO_3)_2$ (4.5 mL, 0.1 M) and btc (3 mL, 0.1 M) aqueous solution were added to 150 mL of ethanol/DI water mixture (v/v =1:1) under vigorous stirring at room temperature for 10 min. The product was collected by centrifugation (10000 rpm, 15 min) and washed with ethanol for 3 times to remove the unreacted reagents. The final product was dried under vacuum and stored as powder at room temperature.

The pluronic F127 was used to modify MOF-199 NPs. The MOF-199 NPs (1 mg/mL in ethanol) and pluronic F127 (10 mg/mL in ethanol) were mixed in ethanol under vigorous stirring for 20 min. Subsequently, the solvent was removed by rotary evaporation. The dried mixture was added to DI water to prepare aqueous dispersion of

^{F127}MOF-199 NPs.

GSH-responsiveness of ^{F127}MOF-199 NPs

To study GSH-responsive degradation, ^{F127}MOF-199 NPs (200 μg/mL) was dispersed in PBS (pH = 7.4) with and without GSH (10 mM) at 37 °C under constant shaking. At the given time (0, 10, 30, 60 and 180 min), the mixture was withdrawn to measure hydrodynamic diameter, and then centrifuged for TEM detection.

To quantify the released Cu^{2+} from GSH-responsive degradation of ^{F127}MOF-199 NPs, the extracted mixture at the given time (0, 10, 30, 60 and 180 min) were digested with aqua regia, and then measured by ICP-MS.

For extracellular GSH depletion study, DTNB was used as the probe. ^{F127}MOF-199 NPs solution of different concentrations (0, 5, 10, 20, 40, 80 μg/mL) were mixed with GSH (10 mM) for 30 min at 25 °C, respectively. Then, DTNB (2.5 mg/mL) was added to the above solution under stirring for 5 min. The mixture was centrifuged to remove the unreacted NPs, and UV-vis spectrophotometer was used to measure the absorbance at 412 nm.

Sulfidation process of ^{F127}MOF-199 NPs

NaHS was used to stimulate endogenous H_2S for sulfidation process of ^{F127}MOF-199 NPs. 1 mL of 6 mM NaHS solution was added to 1 mL of 400 μg/mL ^{F127}MOF-199 NPs solution under magnetic stirring. The UV-vis absorption spectra were measured at different reaction time (0 min, 10 min, 2 h, 4 h, 6 h, 8 h, 10 h, 12 h, 24 h). The final product was collected after 24 h of reaction and then centrifugated (12000 rpm, 10 min) for further characterizations (TEM, XPS, XRD).

In vitro photothermal performance of the sulfidation product

The sulfidation products were collected by centrifugation for the study of photothermal performance. The heating curves were measured by the 1064 nm laser irradiation (1 W/cm^2, 10 min) of the sulfidation products with different concentrations (0, 25, 50, 100, 150, 200 μg/mL), and the irradiation of the sulfidation products (200 μg/mL) under

different powers (0.4, 0.8, 1, 1.4 W/cm^2, 10 min), respectively. A FOTRIC 225 thermal camera was used to record the temperature change, and the corresponding heating curves were recorded. The photothermal conversion efficiency (η) was calculated using the following equation:

$$\eta = \frac{hS(T_{Max} - T_{Sur}) - Q_{Sys}}{\mathrm{I}(1 - 10^{-A})}$$

where h is a heat transfer coefficient, S is the surface area of the container, T_{Max} is the equilibrium temperature, T_{Surr} is the temperature of the surroundings, Q_{Sys} is the heat dissipated from the light absorbed by the container itself, I is the laser power density (1 W/cm^2), and A is the absorbance at 1064 nm.

For photothermal stability study, the sulfidation product of 200 μg/mL was irradiated with 1064 nm laser (1 W/cm^2) for 10 min and then cooled to room temperature. The above step was repeated for 3 times, and the temperature was recorded.

Hydroxyl radical (·OH) generation of the sulfidation product

The TMB (ROS probe), TPA (•OH probe), and ESR were used study •OH generation of the sulfidation product. For TMB assay, 200 μL of 500 μg/mL sulfidation product solution was mixed with 25 μL of 5 mM TMB (dissolved in DMSO) at pH 7.4, and then 25 μL of 10 mM H_2O_2 was added. For the groups with irradiation, the 1064 nm laser of 1 W/cm^2 was applied for 10 min. Subsequently, UV-vis spectrophotometer was used to measure the absorbance at 650 nm.

For the TPA test, 200 μL of 500 μg/mL sulfidation product solution was mixed with 25 μL of 10 mM H_2O_2 at pH 7.4. For the groups with irradiation, the 1064 nm laser of 1 W/cm^2 was applied for 10 min. Then 25 μL of 5 mM TPA was added. After 10 min of processing, the fluorescence signal from 360 to 600 nm by 312 nm excitation was detected.

Furthermore, the generation of •OH was estimated with ESR analysis. 200 μL 500 μg/mL sulfidation product solution was mixed with 20 μL 10 mM H_2O_2 at pH 7.4. For the groups with irradiation, the 1064 nm laser of 1 W/cm^2 was applied for 10 min. The obtained solution supernatant was used for ESR analysis.

Cell culture

The human hepatocellular carcinoma (L02) cells, mouse embryonic (3T3) cells and mouse colon cancer cells (CT26) cells were purchased from Shanghai Institute of Biochemistry and Cell Biology, Chinese Academy of Sciences. All cells were cultured in DMEM or 1064 medium containing 10% heat-inactivated fetal bovine serum (FBS, Gibco) and 1% double antibody (penicillin/streptomycin) solution. The cells were maintained in a constant temperature incubator at 37 °C in an atmosphere of 5% CO_2.

Cellular uptake study

The cellular uptake of ^{F127}MOF-199 NPs was studied by labelling with Cy5.5 and analyzed by flow cytometry. The fluorescence labeling of MOF-199 NPs was performed by directly mixing of MOF-199 NPs with Cy5.5, and then modified by pluronic F127 use the method mentioned above. CT26 cells were seeded into 6-well plates at a density of 1×10^5 cells/well and cultured for 24 h. Then Cy5.5 labelled ^{F127}MOF-199 NPs (200 μg/mL) incubated with the cells for pre-determined time. The cells were washed with PBS, and collected for flow cytometry analysis.

Cytotoxicity evaluation

Cell viability was measured by CCK assay. CT26, L02 and 3T3 cells were seeded on 96-well plates at a density of 8000 cells/well and incubated for 24 h. The medium was then replaced with fresh medium containing ^{F127}MOF-199 NPs with different concentrations (0, 25, 50, 100, 150, 200 μg/mL) and incubated for 24 h. The culture medium was changed and cell viability was evaluated by standard CCK-8 assay. To study the effect of laser irradiation on cytotoxicity, the irradiation (1064 nm laser, 1 W/cm^2, 5 min) was applied after 12 h of incubation with different formulations, followed by incubation for another 12 h. Afterwards, the cell viability was evaluated by CCK-8 assay.

To further examine weather cuproptosis plays an important role in ^{F127}MOF-199 NPs-induced cell death, CT26 cells were pre-treated with cuproptosis inhibitor Rotenone (0.1 μM) for 10 h. Then various concentrations (0, 25, 50, 100, 150, 200 μg/mL) of

^{F127}MOF-199 NPs were added. The irradiation (1064 nm laser, 1 W/cm^2, 5 min) was applied after 12 h of incubation with different formulations, followed by incubation for another 12 h. Afterwards, the cell viability was evaluated by CCK-8 assay.

Intracellular GSH depletion

The intracellular GSH depletion was studied by using DTNB as the probe. CT26 cells were seeded into 6-well plates at a density of 1×10^5 cells/well and incubation for 24 h. The medium was replaced with fresh medium containing PBS, ^{F127}MOF-199 NPs (200 μg/mL). After incubation for 6 h, the groups with irradiation were exposed with 1064 nm laser (1 W/cm^2) for 5 min, followed by incubation for another 6 h. Afterwards, intracellular GSH depletion levels were detected using the glutathione reductase assay kit (DTNB method) according to the manufacturer's instructions.

Intracellular H_2S depletion

CT26 cells were seeded into confocal dishes. When 80% density was reached, the medium was replaced with fresh medium containing PBS or ^{F127}MOF-199 NPs (200 μg/mL). After incubation for 3 h, the groups with irradiation were exposed with 1064 nm laser (1 W/cm^2) for 5 min, followed by incubation for another 3 h. Then, the cells were washed by PBS, stained with WSP-1, and detected by confocal laser scanning microscope (CLSM).

Intracellular ROS generation

CT26 cells were seeded into confocal dishes at a density of 1×10^4 cells/well. When 80% density was reached, the cells were treated with PBS or ^{F127}MOF-199 NPs (200 μg/mL). After incubation for 3 h, the groups with irradiation were exposed with 1064 nm laser (1 W/cm^2) for 5 min, followed by incubation for another 3 h. The cells were washed with PBS, stained with DCFH-DA, and then detected by CLSM.

Observation of mitochondrial damage

CT26 cells were seeded into confocal dishes at a density of 1×10^4 cells/well. When 80%

density was reached, the cells were treated with PBS or ^{F127}MOF-199 NPs (200 μg/mL). After incubation for 3 h, the groups with irradiation were exposed with 1064 nm laser (1 W/cm^2) for 5 min, followed by incubation for another 3 h. The cells were washed with PBS, stained with DAPI and MitoTracker Deep Red FM, and then detected by CLSM.

Cell morphology observation

The cell morphology was observed by inverted microscopy. CT26 cells were planted into 6-well plates and incubated for 24 h. The medium was replaced with fresh medium containing PBS and ^{F127}MOF-199 NPs (200 μg/mL). After incubation for 6 h, the groups with irradiation were exposed with 1064 nm laser (1 W/cm^2) for 5 min, followed by incubation for another 6 h. Then the cell morphology was observed by inverted microscopy.

Western blot assay

CT26 cells were seeded into 6-well plates at a density of 1×10^5 cells/well and cultured for 24 h. For protein analysis of GSDME, DLAT, FDX1, LIAS, the cells were treated with different formulations, respectively. After incubation for 12 h, the groups with irradiation were exposed with 1064 nm laser (1 W/cm^2) for 5 min, followed by incubation for another 12 h. Then, the cells were washed with PBS and lysed with RIPA lysis buffer including PMSF (1 mM). Cell lysates were collected, protein concentration was quantified by the bicinchoninic acid (BCA) method. After boiled in loading buffer, aliquots of each protein sample were separated on 10% SDS-PAGE gel and transferred onto the PVDF membranes. The membranes were blocked with QuickBlock™ Blocking Buffer (TBSTw) for 30 min, then incubation with primary antibodies (GSDME, DLAT, FDX1, LIAS or α-Tubulin) overnight at 4 °C and secondary antibody for 1 h at room temperature, the PVDF membranes were washed with the washing buffer and detected using a chemiluminescence system (Bio-Rad, USA).

Caspase-3 assay

CT26 cells were seeded into 6-well plates at a density of 1×10^5 cells/well and cultured for 24 h. The medium was replaced with fresh medium containing PBS, ^{F127}MOF-199 NPs (200 μg/mL). After incubation for 12 h, the groups with irradiation were exposed with 1064 nm laser (1 W/cm^2) for 5 min, followed by incubation for another 12 h. Then intracellular caspase-3 activation was detected by flow cytometry using GreenNuc™ Live Cell Caspase-3 Activity Assay Kit according to the manufacturer's instructions. The quantitative analysis was measured by Image J software.

ATP release assay

CT26 cells were seeded on 96-well plates and cultured for 24 h. The medium was replaced with fresh medium containing PBS, ^{F127}MOF-199 NPs (200 μg/mL) and. After incubation for 6 h, the groups with irradiation were exposed with 1064 nm laser (1 W/cm^2) for 5 min, followed by incubation for another 6 h. Then released ATP was detected using ATP assay kit according to the manufacturer's instructions.

Immunofluorescent staining

CT26 cells were seeded into confocal dishes at a density of 1×10^4 cells/well. When 80% density was reached, the medium was replaced with fresh medium containing PBS, ^{F127}MOF-199 NPs (200 μg/mL), after incubation for 12 h, the groups with irradiation were exposed with 1064 nm laser (1 W/cm^2) for 5 min, followed by incubation for another 6 h. The cells were sequentially fixed with 4% paraformaldehyde for 30 min, incubated with the antibody (anti-cytochrome c/anti-DLAT/anti-HMGB1/anti-CRT) at 4 °C overnight. Subsequently, they were treated with FITC labeled anti-mouse secondary antibody at room temperature for 1 h, stained with Hoechst 33342 for 15 min, and then imaged by CLSM.

In Vitro ELISA Measurement.

The 80% density CT26 cells were cocultured with fresh medium containing PBS, ^{F127}MOF-199 NPs (200 μg/mL), after incubation for 12 h, the groups with irradiation

were exposed with 1064 nm laser (1W/cm^2) for 5 min, followed by incubation for another 16 h. The upper liquid was collected for measurement of the extracellular IL-18 and IL-1β using ELISA Kit, in accordance with the requirement specified in the manufacturer's instruction. In brief, the treated cell culture medium (100 μL) was added into a precoated microplate, followed by incubating with the detection antibodies at 25 °C for 2 h. The plates were washed by washing buffer for 6 times, added HRP-linked streptavidin for 45 min. Then plates were also washed by washing buffer for 6 times, and added TMB substrate for 25 min. After adding stop solution, read at 450 nm using a microplate reader.

Bone-marrow-derived dendritic cells (BMDCs) maturation in vitro

To investigate dendritic cells (DCs) maturation in vitro, BMDCs were collected from the bone marrow of female C57BL6 mice (3~8 weeks old), and then further cultured for 1 week. CT26 cells were seeded into confocal dishes at a density of 1×10^4 cells/well. When 80% density was reached, the medium was replaced with fresh medium containing PBS, ^{F127}MOF-199 NPs (200 μg/mL), After incubation for 12 h, the groups with irradiation were exposed with 1064 nm laser (1 W/cm^2) for 5 min, followed by incubation for another 12 h. Subsequently, BMDC was cocultured with CT26 cells supernatant after different treatments. The anti-CD11c-FITC (Clone: N418), antiCD80-PE (Clone: 16-10A1) and anti-CD86-APC (Clone:GL-1) antibodies were used to label BMDCs, and the maturation of DCs was examined by flow cytometry analysis.

Animal model

Female BALB/c mice (4~5 weeks old) were purchased from Nanjing Keygen Biotech Co., Ltd. The CT26 tumor models were established by subcutaneously injecting of CT26 cells (1×10^6, 0.1mL cells in PBS) into the right flanks. When tumor volume reached ~150 mm^3, the mice were subjected to in vivo experiments. All animal experiments were performed in accordance with the protocols and approved by the Experimental Animal Ethics Committee of Nanjing Surui Medical Technology Co., Ltd.

In vivo fluorescence imaging

To trace the intratumoral retention behavior of ^{F127}MOF-199 NPs, the MOF-199 NPs were firstly loaded with Cy5.5, and then modified by pluronic F127. Fluorescence imaging was carried out when the tumors reached ~200 mm^3. The Cy5.5-labeled ^{F127}MOF-199 NPs (20 mg/kg) was intratumorally injected by a microsyringe into CT26-bearing mice. The mice were imaged at different time points with small animal fluorescence imaging system.

Biosafety Evaluation

Hemolysis test was used to evaluate blood compatibility of ^{F127}MOF-199 NPs. The mice blood was collected, and the red blood cells were acquired by centrifugation (1400r, 7 min). Then, the blood cells were washed with PBS and diluted to 2% concentration, which were incubated with ^{F127}MOF-199 NPs of different concentrations (0, 25, 50, 100, 150, 200 μg/mL) at 37 °C for 4h. The 0.2% Triton X-100 was used as a positive control. The hemolysis rates were evaluated by measuring the absorbances of the mixture at 540 nm.

The serum biochemical analysis was performed after 14 days of tumor therapy. The female BALB/c mice were given intratumoral injections with different formulations: PBS, PBS+L, ^{F127}MOF-199 (20 mg/kg), ^{F127}MOF-199+L. After 14 days of treatments, the blood in each group was collected and then used for serum biochemical analysis. In addition, the major organs were also collected for hematoxylin and eosin (H&E) staining.

In vivo antitumor therapy

The CT26-bearing mice were randomly divided into four groups (n = 5): (1) control (PBS), (2) control+L (PBS+L), (3) ^{F127}MOF-199 (20 mg/kg), (4) ^{F127}MOF-199+L. Different formulations were intratumoral injected into mice every 2 days. The 1064 nm laser irradiation (1 W/cm^2, 5 min) was applied after 12h of postinjection. The tumor size and mouse body weight were measured every 2 days. The tumor volume was calculated based on the equation: volume = (tumor length) × (tumor width)^2/2. After 14

days of treatments, all mice were sacrificed and tumors were collected for H&E staining, immunofluorescence staining (DLAT, CD8), and immunohistochemical staining (Ki-67, TUNEL, LIAS).

Analysis of immune cells

The in vivo immune activation assay was performed on day 14 after the sacrifice of mice. The axillary lymph nodes and spleens were harvested, and the single cell suspensions were obtained after digestion according to the standard protocol. For the analysis of mature DCs, cells were stained by PerCP/Cyanine5.5 anti-mouse CD45 (Biolegend:103132), PE/Cyanine7 anti-mouse CD11c (Biolegend:117317), APC anti-mouse CD80 (Biolegend:104714) and PE anti-mouse CD86 (Biolegend:105008) according to the supplier's protocol. For the analysis of T cells, cells were stained by PerCP/Cyanine5.5 anti-mouse CD45 (Biolegend:103132), APC anti-mouse CD3 (Biolegend:155606), FITC anti-mouse CD4 (Biolegend:100510), PE anti-mouse CD8a (Biolegend:100707) according to the supplier's protocol. All samples were analyzed by flow cytometry.

For tumor slices analysis, different groups were stained with DAPI, fluorochrome-conjugated anti-mouse CD8 antibody, and fluorochrome-conjugated anti-mouse CD4 antibody, and then analyzed by fluorescence microscope.

Cytokine analysis

For cytokine analysis, serum samples of different groups were collected and diluted before measurements. TNF-α, IFN-γ, IL-1β, IL-18 were detected using ELISA kits according to the manufacturer's protocol. Use the method mentioned above, just replacing the cell culture medium (100 μL) with the serum and assay buffer.

Statistical analysis

All the data were expressed as the mean ± SD, and data analyses were performed using the Prism software. The statistical analysis was performed using one-way ANOVA and Tukey's tests.

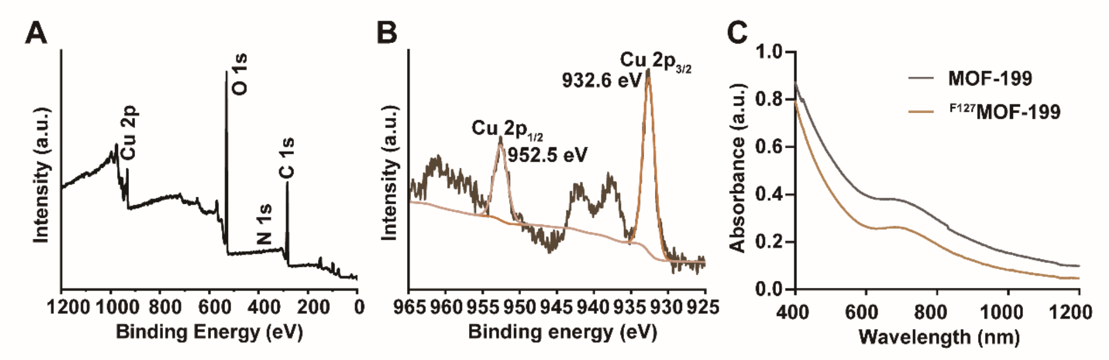

Figure S1. (A) XPS spectrum for a survey scan and (B) high-resolution XPS spectrum of Cu 2p of ^{F127}MOF-199 NPs. (C) UV-vis-NIR absorption spectra of MOF-199 NPs and ^{F127}MOF-199 NPs.

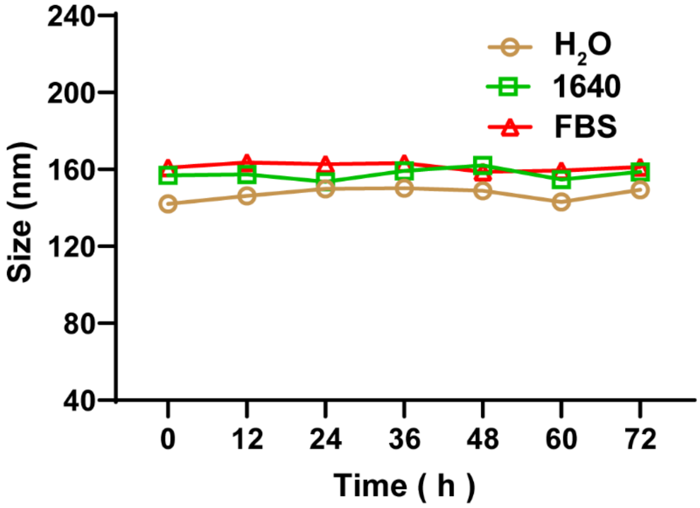

Figure S2. Stability study of F127MOF-199 NPs by measuring hydrodynamic diameter of F127MOF-199 NPs dispersed in different biological media (H_2O, 1640, FBS) for 3 days.

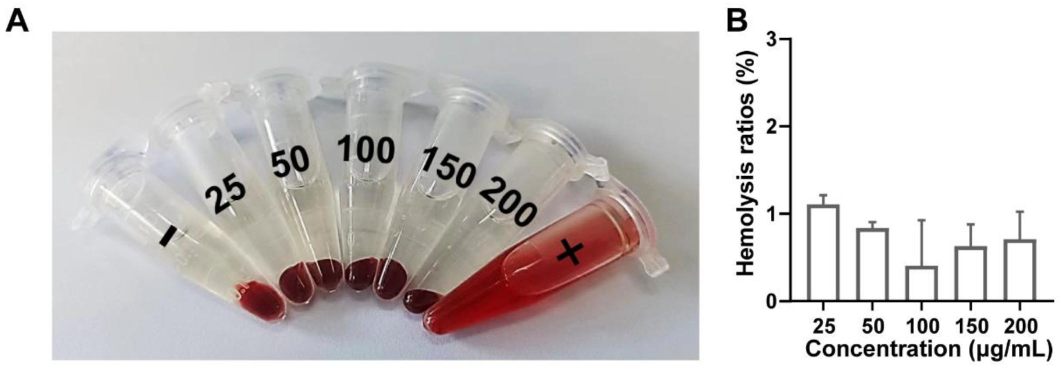

Figure S3. (A) Hemolysis pictures of ^{F127}MOF-199 NPs with different concentrations, Triton X-100 as the positive control, and 0.9% saline as the negative control. (B) Hemolysis ratio analysis of ^{F127}MOF-199 NPs with different concentrations.

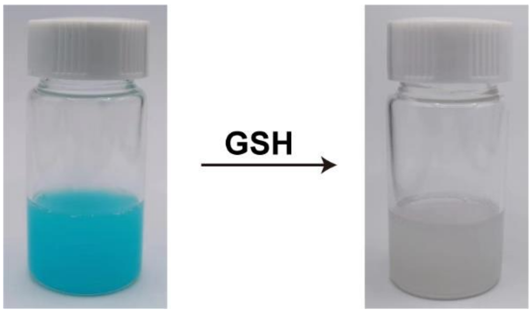

Figure S4. The color change of ^{F127}MOF-199 NPs solution before and after the addition of GSH.

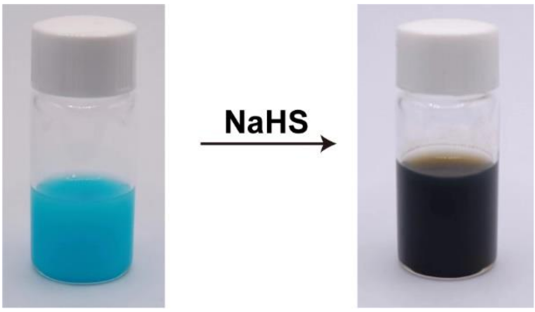

Figure S5. The color change of ^{F127}MOF-199 NPs solution before and after the addition of NaHS.

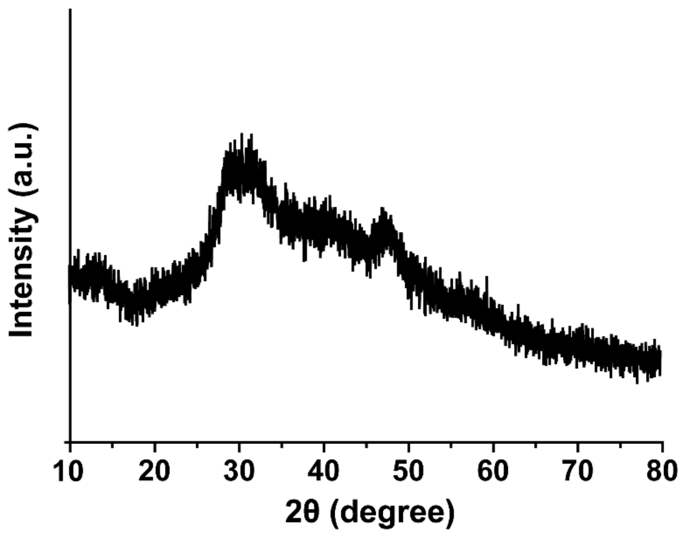

Figure S6. The X-ray diffraction (XRD) patterns of ^{F127}MOF-199 NPs after sulfidation.

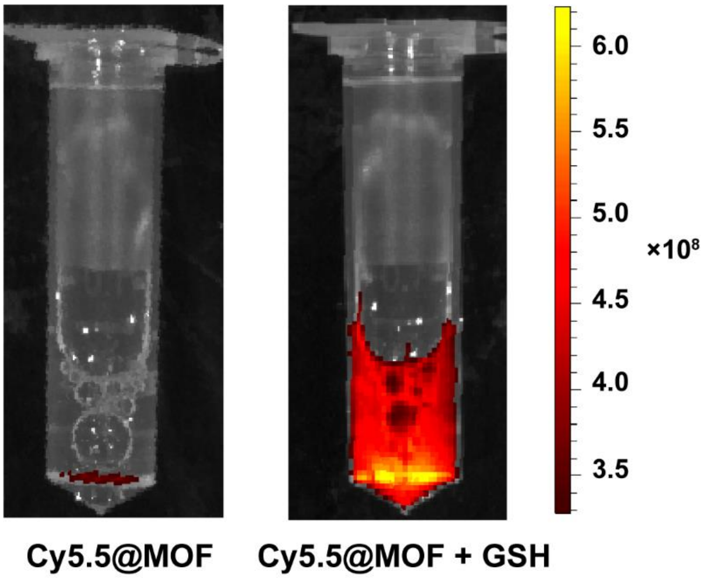

Figure S7. The fluorescence imaging of Cy5.5-labelled ^{F127}MOF-199 NPs before (Cy5.5@^{F127}MOF-199) and after (Cy5.5@^{F127}MOF-199+GSH) the addition of GSH.

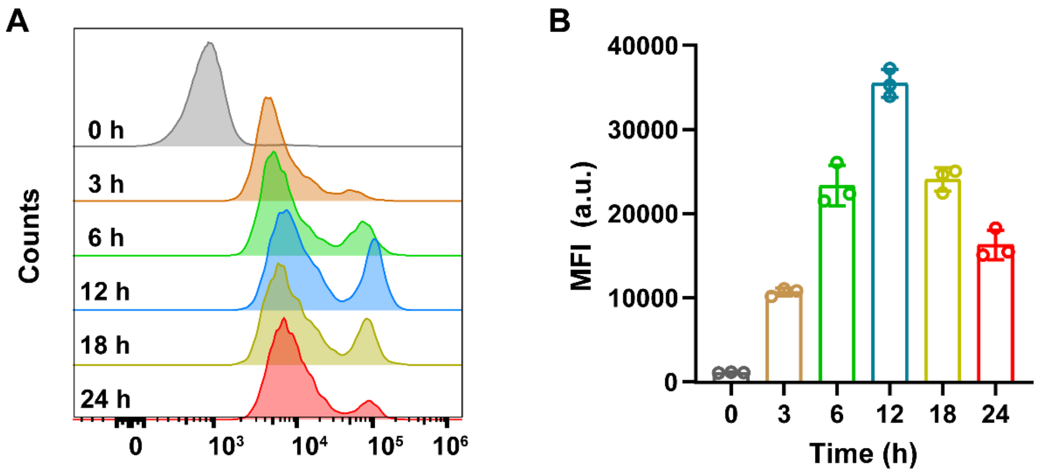

Figure S8. (A) The flow cytometry and (B) the corresponding mean fluorescence intensity (MFI) analysis of cellular uptake after incubation of Cy5.5-labelled ^{F127}MOF-199 NPs with CT26 cells for different time.

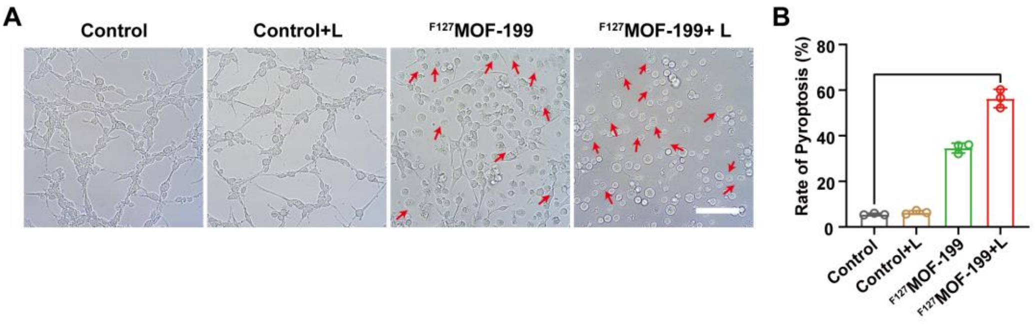

Figure S9. (A) Morphology of CT26 cells after different treatments in large scale (Scale bar = 50 μm). The red arrows point out the pyroptosis cells. (B) The analysis of the ratio of pyroptotic cells after different treatments.

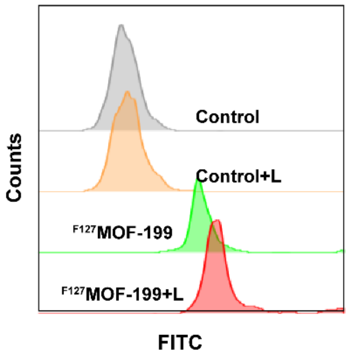

Figure S10. The intracellular caspase-3 activation by flow cytometry analysis.

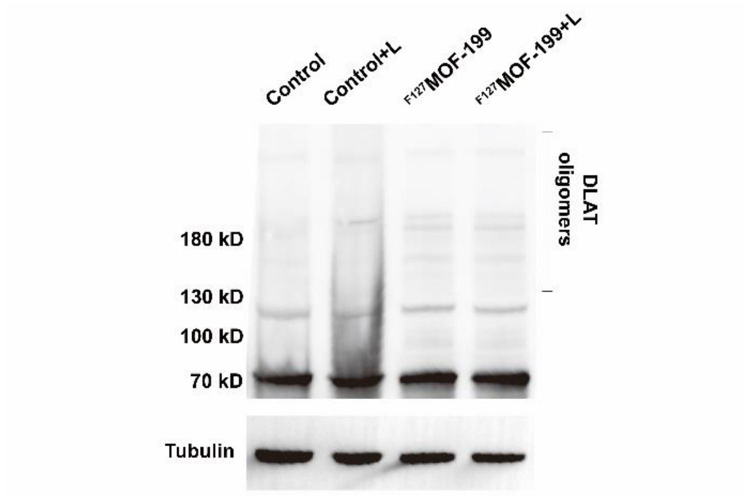

Figure S11. Western blot analysis of the expressions of DLAT oligomers.

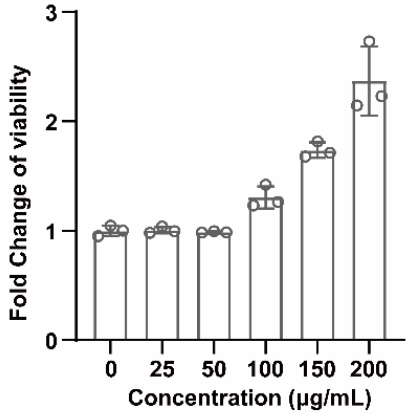

Figure S12. Fold change in viability of CT26 cells treated with ^{F127}MOF-199+L with cuproptosis inhibitor (Rotenone 0.1 μM) relative to that of ^{F127}MOF-199+L without cuproptosis inhibitor.

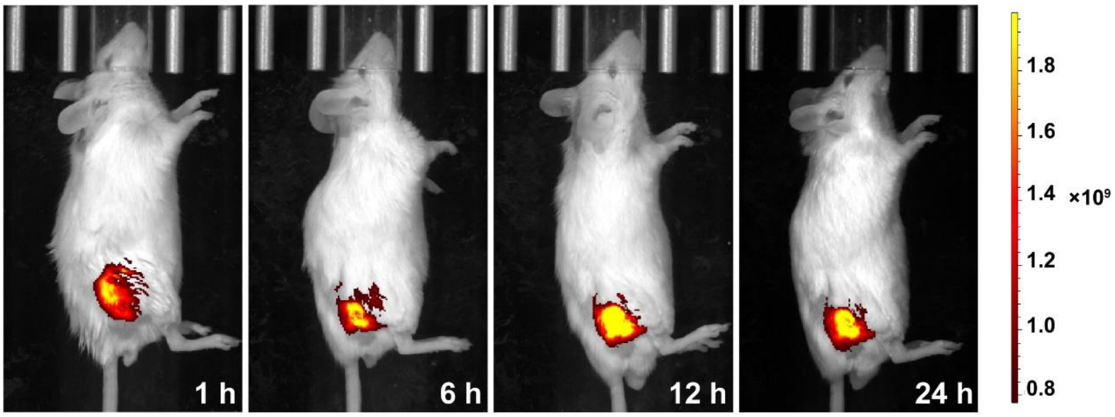

Figure S13. The fluorescence imaging of CT26-bearing mice at different time points after intratumoral injection of Cy5.5 labeled ^{F127}MOF-199.

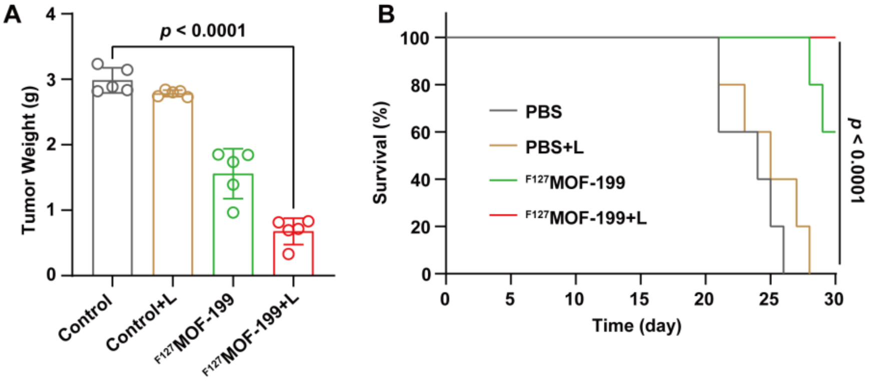

Figure S14. (A) The weight of the excised CT-26 tumors after different treatments. (B) The survival curves of the CT26-bearing mice receiving different treatments.

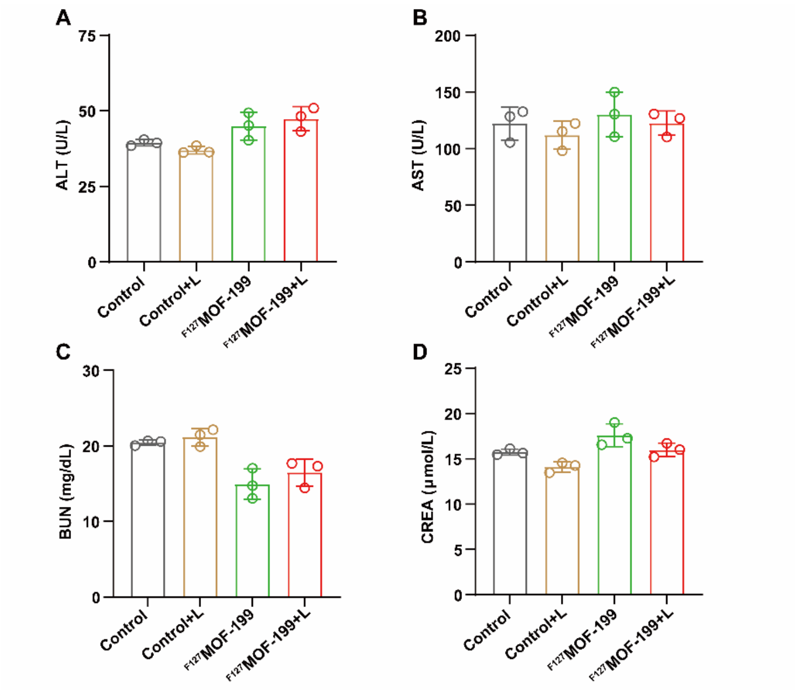

Figure 15. Analysis of blood biochemical indexes of (A-B) the liver (ALT, AST) and (C-D) the kidney (BUN, CRE) at the end of the antitumor study.

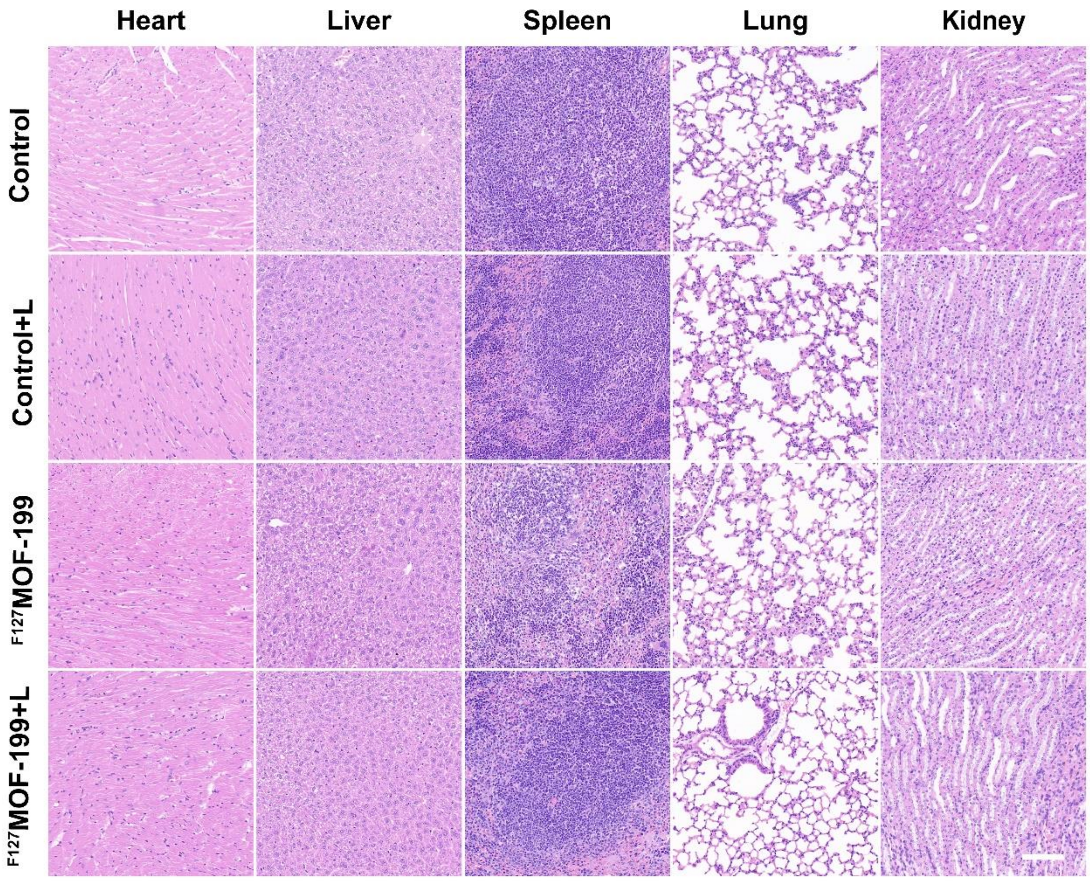

Figure S16. H&E staining of the major organs (heart, liver, spleen, lung and kidney) after different treatments (scale bar = 100 μm).

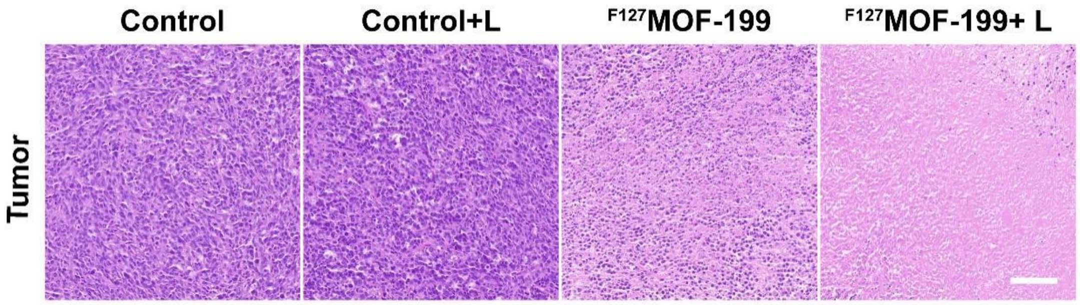

Figure S17. H&E staining of the tumor after different treatments (scale bar = 100 μm).

References:

[1] Q. Liu, L. N. Jin, W. Y. Sun, *Chem. Commun.* **2012**, 48, 8814.
